# Cytosolic Crowding Drives the Dynamics of Both Genome and Cytosol in *Escherichia coli* Challenged with Sub-lethal Antibiotic Treatments

**DOI:** 10.1016/j.isci.2020.101560

**Published:** 2020-09-15

**Authors:** Michal Wlodarski, Leonardo Mancini, Bianca Raciti, Bianca Sclavi, Marco Cosentino Lagomarsino, Pietro Cicuta

**Affiliations:** 1 Biological and Soft Systems, Cavendish Laboratory, University of Cambridge, Cambridge, UK; 2 Laboratory of Biology and Applied Pharmacology (UMR 8113 CNRS), École Normale Supérieure, Paris-Saclay, France; 3 Laboratory of Computational and Quantitative Biology (UMR 7238 CNRS), Sorbonne Université, Paris, France; 4 IFOM Foundation FIRC Institute of Molecular Oncology Foundation, Milan 20139, Italy; 5 Dipartimento di Fisica and I.N.F.N., Università degli Studi di Milano, Via Celoria 16, 20133 Milano, Italy

**Keywords:** Chromosome Organization, Cell Biology

## Abstract

In contrast to their molecular mode of action, the system-level effect of antibiotics on cells is only beginning to be quantified. Molecular crowding is expected to be a relevant global regulator, which we explore here through the dynamic response phenotypes in *Escherichia coli*, at single-cell resolution, under sub-lethal regimes of different classes of clinically relevant antibiotics, acting at very different levels in the cell. We measure chromosomal mobility through tracking of fast (<15 s timescale) fluctuations of fluorescently tagged chromosomal loci, and we probe the fluidity of the cytoplasm by tracking cytosolic aggregates. Measuring cellular density, we show how the overall levels of macromolecular crowding affect both quantities, regardless of antibiotic-specific effects. The dominant trend is a strong correlation between the effects in different parts of the chromosome and between the chromosome and cytosol, supporting the concept of an overall global role of molecular crowding in cellular physiology.

## Highlights

• Motility of loci positioned on different parts of the chromosome is correlated to sub-lethal antibiotic treatments
• Motility changes of chromosomal loci correlate with those of cytosolic aggregates
• Treatment-induced changes to crowding levels in cytosol are consistent with the changes in motility

## Graphical Abstract

**Figure:**
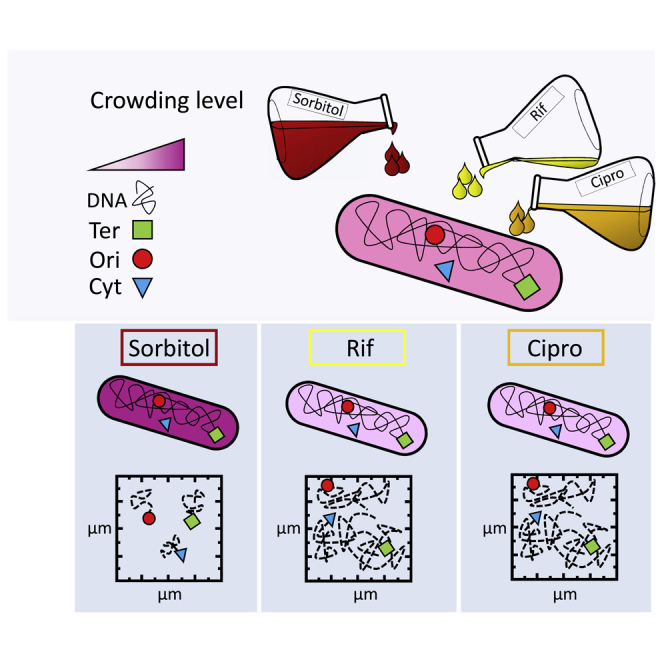

## Introduction

Antibiotic perturbations cause an interplay of physiological responses and specific responses to treatment; in general, this is still an open question, with potential impact in the biomedical field. Although the molecular mechanisms of action of most antibiotics are well known, their effects on the physiology of the cell at a “systems” level remain largely unexplored. For example, we currently cannot predict the precise effects of treatments on gene expression patterns. These changes that affect the cell as a system may be due to physical aspects of the cell state, such as the levels of molecular crowding and the compaction state of the genome (Pelletier et al., 2012). Crowding, for example, may affect both genome compaction and the rates of several biochemical processes, and so these effects potentially cascade into a wide range of cellular behavior (Miermont et al., 2013; Tsai et al., 2019; Oldewurtel et al., 2019).

The nature and dynamics of how a cell responds to perturbation are complex and depend on the antibiotic class (and thus target molecule abundance, Mitosch and Bollenbach, 2014), dose (Greco et al., 1995), exposure time (Michael et al., 2016), affinity to the target molecule (Greulich et al., 2015), as well as bacterial species (Rahal and Simberkoff, 1979; Gupta, 2011), growth phase (Wood et al., 2013), rate (Tuomanen et al., 1986; Brown et al., 1990; Greulich et al., 2015), and environment (Kwon et al., 2010; Mitosch and Bollenbach, 2014). The fact that bacteria can use antibiotic molecules for signaling, for example, to coordinate multicellular processes within a population in antibiotic-induced biofilm growth (Jerman et al., 2005; Hoffman et al., 2005), exemplifies both the complexity and elegance of such responses. The multi-factorial character of bacterial responses to antibiotics brings together a number of distinct (e.g., genetic, metabolic, structural) processes impacting the cell's physiology.

However, exposure to antibiotics also perturbs the global physiological state of the cell. Exposure to an antibiotic may change the expression rate of a specific gene or gene cluster to compensate directly for the drug's action, for example by increasing the number of target protein to compensate for its inhibition or to synthesize machinery to repair antibiotic-induced damage (Lin et al., 2005; Khil and Camerini-Otero, 2002; Shaw et al., 2003). Also, through global changes to the physiology (e.g., effects on the growth rate), antibiotics alter global gene expression patterns (Mitosch and Bollenbach, 2014). The total number of genes affected varies significantly for different antibiotics (Lin et al., 2005). Furthermore, antibiotics have been shown to affect macromolecular composition, including protein-to-DNA ratio (Bollenbach et al., 2009), and the RNAP (Klumpp and Hwa, 2008) and ribosome concentrations (Scott et al., 2010). The concentrations of other key cellular species such as second messengers (e.g., Hoffman et al., 2005) and the alarmone molecule, (p)ppGpp (Dalebroux and Swanson, 2012), were also reported to change, further contributing to global changes in gene expression.

The *E*. *coli* genome is several orders of magnitude longer (∼1.5 mm) than the cell length (typically 1–2 μm), and so it is tightly packaged in the cell volume (Stavans and Oppenheim, 2006; Dame, 2005). It is organized, together with RNA and proteins, into a highly compacted structure called the nucleoid. As reviewed in (Benza et al., 2012), the organization is at various scales, starting at the level of DNA strands, which interact with themselves, RNA, and the nucleoid-associated proteins (NAPs) forming bridges, bends, and loops; then at intermediate length scales, giving rise to supercoiled domains; and then globally in “macro-domains” of several million base pairs. Fluorescence microscopy of tagged genetic sites allows to probe in living cells some of these physical properties, by monitoring the fast local dynamics (fluctuations) of the chromosome (Espeli et al., 2008; Weber et al., 2010a; Javer et al., 2013; Wlodarski et al., 2017; Crozat et al., 2019). If one observes chromosome motions over time intervals longer than approximately 1 minute, they are dominated by segregation and cell growth, which will appear as super-diffusive directed (ballistic) motion (Espeli et al., 2008; Cass et al., 2016). In contrast, displacements at time intervals below 10 s are interpreted as fluctuations in a complex local environment, reflecting “microrheology” properties (Waigh, 2005; Cicuta and Donald, 2007; Wlodarski et al., 2017).

In complex viscoelastic fluids the $MSD\left(\tau \right)=\left\langle {\left(x\left(t+\tau \right)-x\left(\tau \right)\right)}^{2}\right\rangle$, where x is the position of a particle at a given time and the angular brackets reflect a time and/or ensemble average, is often found to follow a scaling *τ*^*α*^, with the exponent 0<*α*<1 indicating sub-diffusion (for diffusion, *α*=1).

The local physical properties of the *E*. *coli* chromosome and cytosol have been probed in a number of experimental studies. Weber et al. first revealed that chromosomal loci move subdiffusively with a power law exponent around *α*∼0.4. Our team also explored these motions, showing in Javer et al. that a small proportion (1%–2%) of chromosomal loci shows seemingly directed ballistic motion (Javer et al., 2014) and that in the presence of this fluorescent tag the amplitude of short time scale chromosomal motion varies for different positions along the genome, with loci located closer to the origin of replication showing larger motions compared with those closer to the terminus of replication (Javer et al., 2013). This is consistent with uneven NAP-binding sites, specifically MatP condensation (Dame et al., 2011; Espéli et al., 2012; Crozat et al., 2019), and enzyme activity distributions along the genome observed previously (Sobetzko et al., 2012).

Similarly to chromosomal loci, cytosolic tracer objects also display subdiffusive motion, with non-trivial size dependence. A recent study by Parry et al. showed that size-calibrated aggregates of cytosolic avian reovirus protein μNS, abbreviated as μNS-GFP (which are foreign to *E*. *coli*) display metabolism-dependent motion, with the difference in the *MSD* between metabolically active and inactive cells increasing strongly with aggregate size (Parry et al., 2014). The same study, and to date the only one, explored the effects of an antibiotic, rifampicin, on cytosol dynamics. A relatively high dose was used to switch off transcription, and this caused a small reduction of mobility in the cytosolic μNS-GFP aggregates. The study measured *MSD* for long lag times (minutes to hours) (Parry et al., 2014). In another study of crowding, hyper-osmotic shock conditions of >0.28 osmol caused a decrease in the cytosol dynamics (quantified as change in the diffusion coefficient of GFP). The change was proportional to the magnitude of the osmotic upshift (Konopka et al., 2006). It is an open question which (or both, jointly) of the chromosome or the crowded cytosol is causing the complex dynamics observed in the other. Consequently, it is valuable to measure both motions under the same cell perturbations.

**Figure 1:**
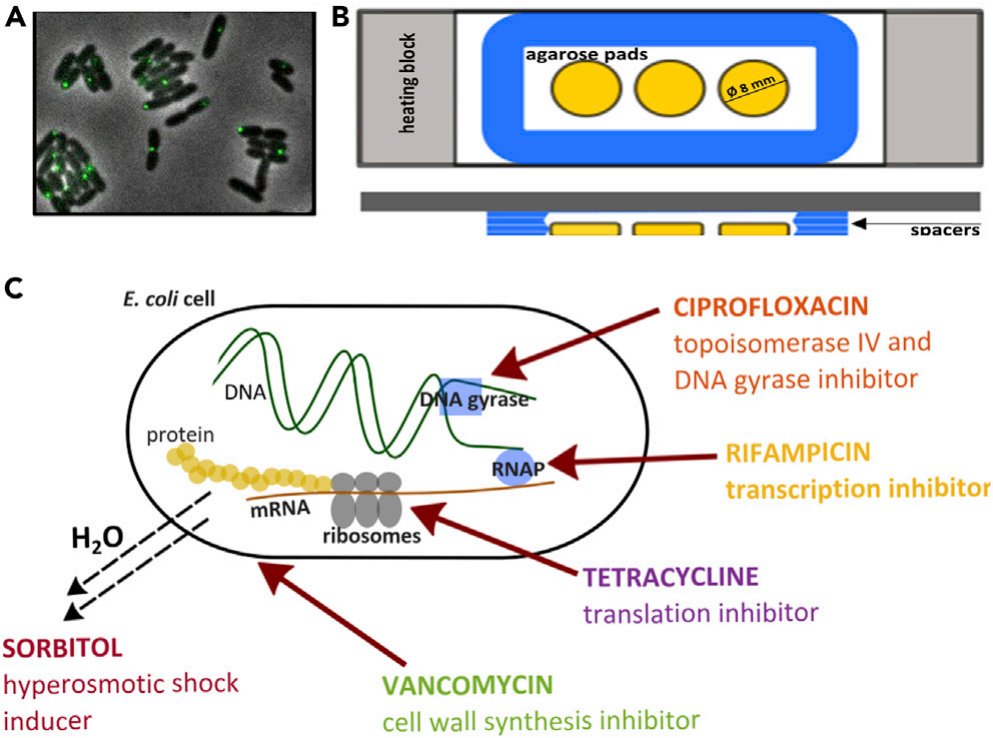
Experimental Approach Chromosomal and cytosolic markers are tracked in each cell, under treatments with antibiotic and crowding agents. The trajectories are quantified through the mean square displacement (*MSD*) of the fluorescent tracer, either chromosomal or cytosolic, following the methods of Wlodarski et al. (2017). All *MSD* are well described by power laws of the time interval τ, *τ*^*α*^. One expects the *MSD* amplitude to be inversely proportional to the viscoelastic resistance of the surrounding medium. Chromosomal Ori2 and Ter3 loci explore space slower than the cytosolic μNS aggregates. (A) Example of phase contrast image of live *E*. *coli* cells, overlayed to the fluorescence channel showing chromosomal loci (*green*). (B) The schematic top and side views show how the samples are contained for live imaging: agarose pads (*yellow*) are sealed between a coverslip and a glass slide with spacers (*blue*) and sit on a heating block (*gray*) for temperature control. (C) We explore sub-lethal doses of four antibiotics, chosen from four major antibiotic classes, and a hyperosmotic shock-inducing agent, sorbitol. The antibiotics are chosen to have very different targets: DNA replication (ciprofloxacin), transcription (ciprofloxacin and rifampicin), translation (tetracycline and chloramphenicol), and cell wall synthesis inhibitors (vancomycin).

In this work, we investigate response phenotypes by characterizing the effects of sub-lethal doses of clinically important antibiotics and of sorbitol (a hyperosmotic shock inducer) on short (<15 s) timescale chromosome and cytosol dynamics in *E*. *coli* grown in standard growth conditions on agarose pads (Figures 1A and 1B). To enable long (several hours) measurements on the same cells, as critical for investigating the effects of antibiotics, it is essential to use the data-correction method for dynamics that we developed previously (Wlodarski et al., 2017); this accounts for both marker photo-bleaching and marker size effects. We find that most tested treatments cause small effects on mobility, coherently for both chromosomal loci and cytosolic aggregates. We also find that different antibiotics have different effects on genome and cytosol dynamics. We then investigate the mechanistic reasons behind the observed changes in intracellular mobility under antibiotic treatments by assaying macromolecular crowding. As a measure of global crowding we assay cell density using optical readings and estimations of dry mass and refractive index (RI). Our results show a previously unknown robust correlation between the mobility of genetic material and of cytoplasmic particles and intracellular density in the presence of antibiotics.

## Results

### Sub-lethal Antibiotic Treatments Cause Small but Consistent Effects on Genome and Cytosol Dynamics

**Figure 2:**
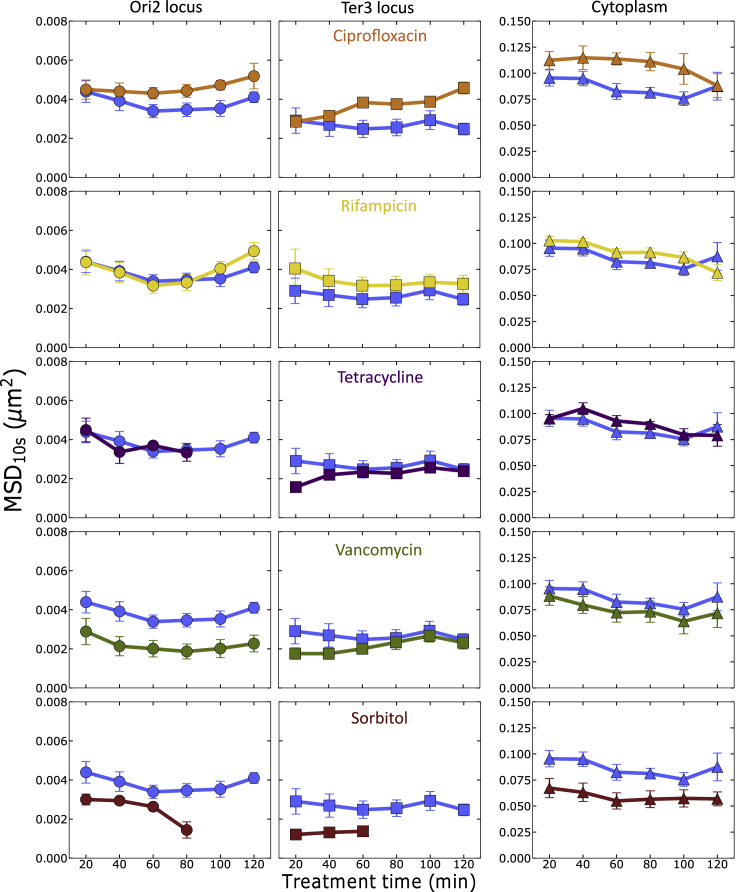
Sub-lethal Antibiotics and Sorbitol Change the Mobility of Both Chromosome and Cytosol There are small but consistent changes to the short timescale mobility *MSD*(10 s) over treatment time (*T*_*treat*_ = 20–120 min) for different antibiotics and sorbitol (in different colors as indicated in the figure), compared with control (*blue* lines) for the marker at the Ori2 locus (*circles*), the Ter3 locus (*squares*), and for the cytoplasmic particle (*triangles*). Data points show the average of the medians from 9 (chromosomal loci) and 6 (particles in cytoplasm) independent replicates. Error bars show the standard error. p Values are given in Table S2 and Supplemental Information.

For an initial comparison of mobility across the effects of treatments we choose the *MSD* at an (arbitrary) lag time of 10 s (τ = 10 s). The results show that in untreated bacteria, mobilities of both chromosomal loci and cytosolic μNS aggregates remain fairly stable throughout the experiments (Figure 2, blue lines on all panels). As expected, the cytosolic aggregates show higher mobilities (*MSD*(10s) ≃ 0.08 μm^2^), whereas chromosomal loci show *MSD*s of an order of magnitude lower at 10-s lags, with Ori2 loci exploring space faster than Ter3 loci (*MSD*(10s) ≃ 4.0×10^−3^ and ≃2.5×10^−3^ μm^2^, respectively).

Bacteria were all first grown in identical conditions (seeMethods), and then delivered on agar pads containing different antibiotics or sorbitol. Antibiotic experiments were performed at sub-lethal doses (∼75% of the minimal inhibitory concentrations [MIC]), determined for each tested strain using a standard agar dilution MIC determination method (Wiegand et al., 2008) (see Methods for details). The bacteria were observed repeatedly for up to 2 h (*T*_*treat*_ = 20–120 min), under treatment with four clinically important antibiotics of distinct classes (and thus different modes of primary action): ciprofloxacin (type II topoisomerase inhibitor), rifampicin (transcription inhibitor), tetracycline (translation inhibitor), and vancomycin (cell wall synthesis inhibitor) as illustrated in Figure 1C. In addition, bacteria were grown on an agar pad with 400 mM sorbitol, capable of inducing a hyperosmotic shock in *E*. *coli* as reported previously (Rojas et al., 2014). Overall, nearly 100,000 chromosome loci tracks were collected across all treatment conditions and measurement times (refer to Table S1 and Supplemental Information for details).

We find that sub-lethal doses of most of the tested antibiotics and sorbitol cause small changes to short timescale (10-s lags) dynamics of both chromosomal loci and cytosolic μNS aggregates (Figure 2). Importantly, the effects are consistent between the three markers and, in most cases, over the entire drug exposure time.

Treatment with ciprofloxacin increases the *MSD* of both Ori2 and Ter3 loci gradually over the treatment time, up to ≃4.0×10^−3^ and ≃2.5×10^−3^ μm^2^ at the final treatment time point (*T*_*treat*_ = 120 min), respectively. Cytosolic μNS aggregates show an increased *MSD* already at the initial time point (*T*_*treat*_ = 20 min) and gradually decrease mobility and reach the control level (≃0.08 μm^2^) at the final treatment time point.

We observe a similar trend for rifampicin, as reported by us recently (Wlodarski et al., 2017). This is a smaller effect than with ciprofloxacin, and especially minute for the Ori2 locus, whose mobility increases up to ≃5.0×10^−3^ μm^2^ only for *T*_*treat*_ > 90 min. Ter3 mobility remains increased fractionally, but consistently, to the level of ≃3.5×10^−3^ μm^2^ across the whole drug exposure time. Effects are also very small for cytosolic μNS aggregates, whose *MSD* remains higher by ∼0.01 μm^2^ throughout the whole treatment time, except for the final time point.

Tetracycline is the only treatment agent tested in this work that does not show consistent effects on the mobility of the three markers. In addition, as translation inhibition causes a decrease in ΔParB-GFP production, the lower amount of fluorescent protein allows photo-bleaching to make all trackable Ori2 loci disappear after 80 min of experiment.

Vancomycin causes a small decrease in *MSD* of both chromosomal loci already at the initial treatment time point (≃3.0×10^−3^ and ≃2.0×10^−3^ μm^2^ for Ori2 and Ter3 loci, respectively) and continues to decrease Ori2 loci mobility down to ≃2.0×10^−3^ μm^2^, whereas Ter3 mobility loci remains relatively constant. Mobility of cytosolic μNS aggregates deceases by ≃0.01 μm^2^ and remains at this level till the end of the experiment. In addition, we observed that vancomycin affected visibly cell morphology causing characteristic bending of cells (Figure S9 and Supplemental Information).

Sorbitol also decreases the *MSD* of both Ori2 and Ter3 loci already at the initial treatment time point (about 20 min into the exposure to sorbitol). Mobility is down to ≃1.5×10^−3^ μm^2^ for both Ori2 and Ter3 loci at final treatment times. Under hyperosmotic shock conditions, and likely also in cells recovered from the shock, gene expression processes may be impaired (van den Berg et al., 2017), resulting in strong marker photo-bleaching and thus inability to collect valid loci tracks after *T*_*treat*_=80 and 60 min for Ori2 and Ter3 loci, respectively. Motility of cytosolic μNS aggregates remains decreased at ≃0.06 μm^2^.

We also observed changes to the scaling exponent α, a result reported recently by Yu et al. (2018) in mechanically compressed *E*. *coli* cells. The antibiotic-induced changes to α observed in this work are much smaller (changes of up to 16% at the shortest *T*_*treat*_, Figure S3) compared with those in compressed cells (change of at least 20% at 20 psi for all markers).

### Directions of Effects Generally Correlate between Chromosomal Ori2 and Ter3 Loci

**Figure 3:**
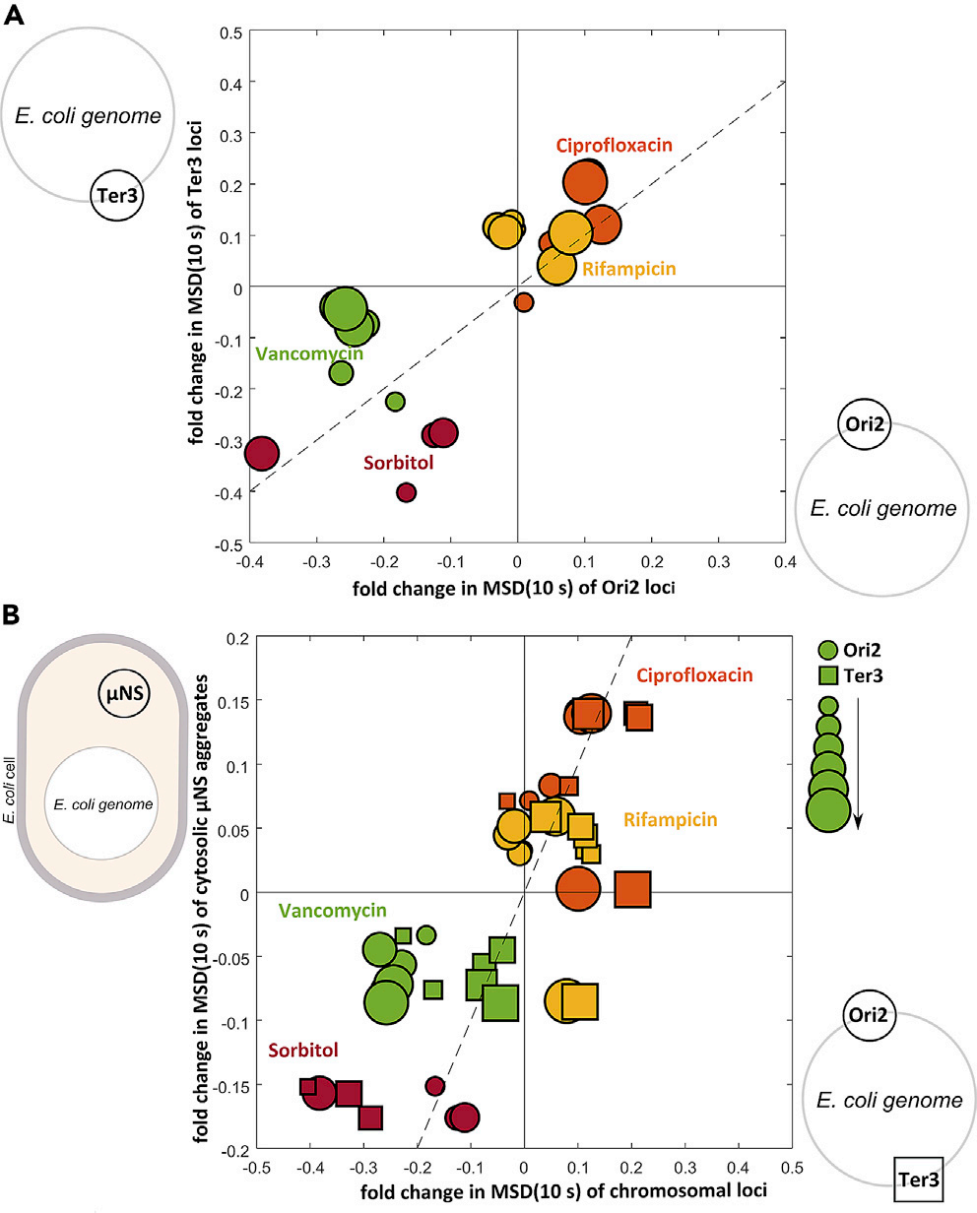
Changes Are Generally Consistent across the Chromosome, and in the Cytosol (A) Directions of effects generally correlate (except for vancomycin) between the chromosomal Ori2 and Ter3 loci. Fold changes in Ori2 versus Ter3 loci mobility, defined as the logarithm of treated-to-control *MSD*(10 s) ratios, are plotted for different treatments (in different colors as indicated in the figure). (B) Direction of effects generally correlates also across chromosomal loci and cytosolic μNS aggregates. Fold changes in chromosomal Ori2 (*circles*) and Ter3 (*squares*) loci versus cytosolic μNS aggregates' mobility, defined as the logarithm of treated-to-control *MSD*(10 s) ratios, are plotted for different treatments (in different colors as indicated in the figure). Plot marker size increases with increasing treatment time (*T*_*treat*_ = 20–120 min). Diagonal dashed lines in both panels represent gradients of unity.

To compare chromosomal responses directly, we considered relative fold changes in mobility, defined as the logarithm of treated-to-control *MSD*(10 s) ratios, for Ori2 and Ter3 loci for individual treatments and treatment times (Figure 3A). The directions of these effects are consistent for the Ori2 and Ter3 chromosomal loci for each of the treatment conditions.

Notably, the initially fast-moving loci, Ori2, increased their mobility less when treated with ciprofloxacin and rifampicin (maximal fold changes +0.12 and +0.08, respectively) when compared with the initially slow-moving loci, Ter3 (maximal fold changes +0.2 and +0.1, respectively). Conversely, the initially slow-moving loci, Ter3, decreased their mobility less when treated with vancomycin (maximal fold change −0.18) when compared with the initially fast-moving loci, Ori2 (maximal fold change −0.28), and showed comparable magnitude in fold change under sorbitol treatment (maximal fold change −0.39 and −0.40 for Ori2 and Ter3, respectively).

### Directions of Chromosomal and Cytosolic Effects Generally Correlate

We can also compare chromosomal and cytosolic responses directly (Figure 3B) considering relative changes in mobility defined again as the logarithm of treated-to-control *MSD*(10 s) ratios for chromosomal Ori2 and Ter3 loci and cytosolic μNS aggregates for individual treatments and treatment times. The directions of responses are generally consistent between chromosomal and cytosolic markers and the magnitude of fold change for chromosome and cytosol dynamics is generally comparable. In fact, most of the responses lie on or near a straight line with gradient equal to unity (Figure 3B, dashed diagonal line). This suggests that changes to the physical properties of cytosol generally correlate both in timing and magnitude with changes to the physical properties of the chromosome. Exceptions to this pattern are the Ori2 loci under vancomycin treatment, and both the chromosomal loci under the sorbitol treatment.

### Detailed Chromosomal Dynamics

**Figure 4:**
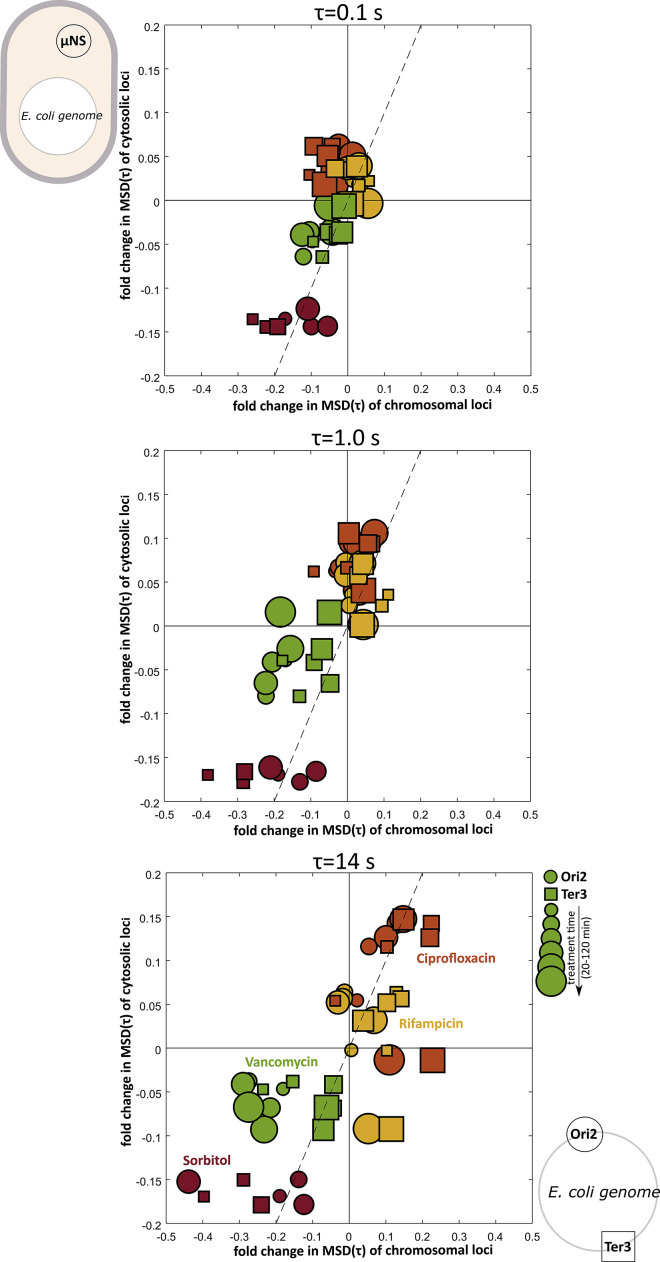
Dynamic Changes with Different Antibiotics and Sorbitol Depend on the Lag Time Fold changes in chromosomal Ori2 (*circles*) and Ter3 (*squares*) loci versus cytosolic μNS aggregates' mobility, defined as the logarithm of treated-to-control *MSD*(10 s) ratios, are plotted for different treatments (in different colors as indicated in the figure) for three lag times (*τ*=0.1, 1.0, and 14 s) representing a range of tested lag times as indicated above individual figures. Plot marker size increases with increasing treatment time (*T*_*treat*_ = 20–120 min). Diagonal dashed lines represent gradient of unity.

We also looked at how dynamics responses evolve as a function of lag time; fold changes in mobility as shown earlier for three arbitrary lag times (*τ*=0.1, 1.0, and 14 s) are plotted in Figure 4. The differences in responses between all tested antibiotics become apparent for *τ*>10 s, with data points for individual treatment conditions forming distinct clouds, especially at longer treatment times. In addition, the difference between the Ori2 and Ter3 response to vancomycin becomes well differentiated only for *τ*>5 s and, for ciprofloxacin and rifampicin, only at longer (*T*_*treat*_>40 min) treatment times. Remarkably, sorbitol follows a different pattern, causing distinct effects for both chromosomal and cytosolic makers already at the shortest tested lag time (*τ*=0.1 s). In addition, the difference between the Ori2 and Ter3 response is apparent already at this lag time and becomes less pronounced but still distinguishable at longer lag times. We compared step-size distributions at *τ*=0.1 s for control (not treated) and sorbitol-treated samples and observed no significant differences (Figure S2).

### Changes in the Dynamics Are Generally Consistent with Intracellular Crowding

We reasoned that as the chromosomal and cytosolic dynamics change proportionally with each other, they are possibly determined by a common factor, and that the changes to the intracellular crowding levels could be an explanation. Having observed cell size changes during treatment that qualitatively recapitulated the trends observed in Figures 4A and 4B (see also Figure S4 and Supplemental Information), we speculated that if cellular mass did not follow the same trends, treated cells might have undergone changes in intracellular density and therefore in crowding levels. In single scattering regimes, that is when cells are sufficiently diluted (Stevenson et al., 2016), OD_600_ linearly correlates with the dry mass of cells (Basan et al., 2015a) (Figure S11 and Supplemental Information). As *E*. *coli* is thought to robustly maintain a constant density across a range of growth conditions and across cell volumes spanning half an order of magnitude (Basan et al., 2015a; Oldewurtel et al., 2019), the relationship between dry mass and OD_600_ remains constant independent of cell size. Although OD_600_ is often considered a synonym of cell number, this is only true for cells with constant cell sizes. As a proxy for intracellular cell density we therefore assayed the relationship between OD_600_ and dry mass in both treated and untreated cells. In contrast to what was observed for cells grown for many generations in constant non-limiting conditions (i.e., steady state) (Basan et al., 2015a), cells treated with sub-lethal concentrations of ciprofloxacin or rifampicin for 1 h exhibited a biomass to OD_600_ ratio 10% lower than that of untreated cells. We observed even more significant changes in the case of sorbitol, although in the opposite direction, with cells showing a biomass-to-OD_600_ ratio larger by about 30% than untreated cells. Our values for untreated cells are overall in agreement with those previously reported in the literature (Ren et al., 2013; Long et al., 2016; Folsom et al., 2014; Basan et al., 2015b; Stevenson et al., 2016).

**Figure 5:**
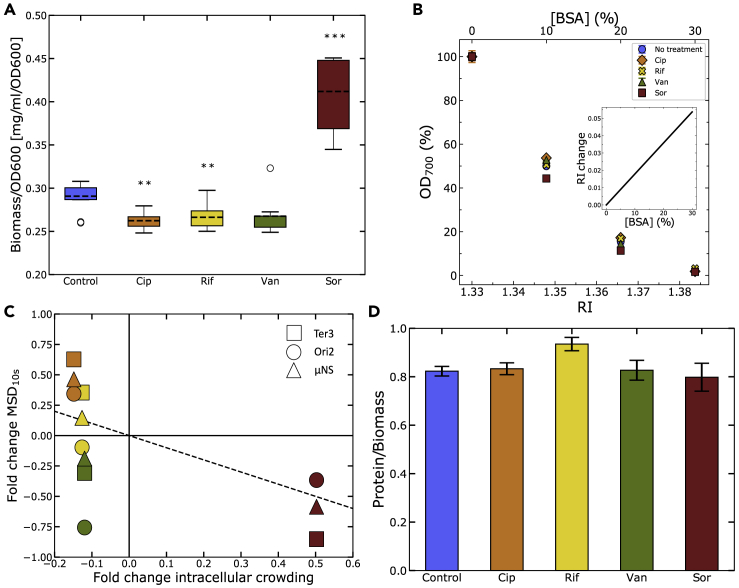
Changes in Cytosol Crowding Levels Are Consistent with Effects on Intracellular Dynamics (A) Biomass to OD_600_ ratio of cells after 1 h of antibiotic treatment. Treatment conditions include: Control (not treated); Cip, ciprofloxacin; Rif, rifampicin; Van, vancomycin; and Sor, sorbitol. Data for each condition were obtained from three biological replicates, performed in triplicates. Statistical significance of the differences with the untreated control was assayed via a t test, obtaining the following p values: Cip, 0.002; Rif, 0.008; Van, 0.052; Sor, 0.0001. (B) Refractive index (RI) of cells measured by comparing with the RI of BSA solutions. Inset: RI change due to dissolved BSA. (C) Scatterplot of the log_2_ of fold change in *MSD*(10 s) after 1 h of treatment versus fold change in crowding levels under the four treatments. The dashed diagonal line represents a gradient of negative unity. (D) Protein contribution to the total biomass for treated and untreated cells. Error bars show the standard error. Error propagation of the error was carried out according to the formula: $SE_{a/b}=\sqrt{{\left(SE_{a}/A\right)}^{2}+{\left(SE_{b}/B\right)}^{2}}$ where *SE*_*a*_ and *SE*_*b*_ are the standard errors of protein and biomass and *A* and *B* are their respective means. Protein quantification data from five biological replicates performed in triplicates with two independent methods. Statistical significance of the changes compared with the control was tested with a t test obtaining the following p values: Cip, 0.917; Rif, 0.267; Van, 0.968; Sor, 0.794.

We then reasoned that the washes necessary for estimating dry mass could potentially cause a certain level of cell lysis to which antibiotic-treated cells may be more susceptible. We tested this by monitoring optical density (OD) after repeated washes finding no sign of lysis (Figure S11B and Supplemental information). We also considered that some cell growth might take place during sample handling. Although in our work antibiotics are used at a sub-inhibitory concentration, they could still slow down the growth rate of treated cells, thereby introducing artifacts in our dry mass measurements. Thus we repeated our measurements by carrying out all the sample handling on ice, obtaining results that are in line with those obtained when the handling was performed at room temperature (Figure S11C and Supplemental Information). (We note that ice could in turn introduce artifacts by altering cell physiology, for example, by inducing the cold shock response). We further reasoned that the changes observed could have also been explained by changes in the RI of the treated cells, which would have influenced the OD readings. OD indeed scales with the ratio between the RI of the cells and the one of the medium, reaching zero OD when the two match (Marquis, 1973; Bateman et al., 1966). We took advantage of this relation to estimate the RI of cells across our treatment conditions. We progressively added BSA, which alters the medium's RI in a known way (Barer and Tkaczyk, 1954; Crespi et al., 2012) (Figure 5B, inset), to cell suspensions until OD became zero. The RI of the BSA solution in which cells had zero OD was therefore equal to the RI of our cells. As suspensions of treated and untreated cells lost OD at similar BSA concentration, we concluded that their RI was not varying significantly and that our biomass-to-OD_600_ values can be interpreted as changes in density.

The changes in density, and thus of crowding levels, caused by the treatments were generally consistent with those observed in the loci and cytoplasmic mobility (Figure 5C) with the exception of vancomycin, which instead showed no correlation. As the antibiotics used in our treatments target different cellular functions, we wondered whether the changes in density were accompanied by changes in macromolecular composition. Given that protein is the most abundant molecular species of the bacterial cell (Bremer and Dennis, 2008), we assayed cells' protein content via two independent colorimetry assays: the Bradford and Biuret assays. Our assays report protein levels for untreated cells that are in good agreement with the literature (You et al., 2013) and did not show any significant change in the relative protein content after treating cells for 1 h with antibiotics or sorbitol, except perhaps a small increase in the case of rifampicin treatment (Figure 5C). Taken together, these findings show that under certain antibiotic treatments (ciprofloxacin and rifampicin) and in certain environmental conditions (hyperosmotic shock), bacteria temporarily lose the ability of regulating their cell density, albeit, at least from the point of view of protein, maintaining the capability of keeping a certain level of control upon the relative abundance of their macromolecular components.

## Discussion

### The Fast Dynamics of Genome and Cytosol at Long (Several Hours) Drug Exposure Times

Many important aspects of how antibiotics affect bacterial physiology remain unknown. Our current challenge is to provide a more holistic picture of antibiotic effects. For example, this question has been approached through DNA microarray studies on global gene expression (Lin et al., 2005). However systems-level physiological responses such as effects on gene regulatory networks and on the macromolecular composition of cells (e.g., the concentration of ribosomes and protein-DNA ratio) remain largely unexplored. Our study provides a complementary viewpoint on the response phenotypes of different antibiotics.

The data treatment procedure we developed (Wlodarski et al., 2017) measures long-term (several hours) changes to marker dynamics, accounting for marker photo-bleaching and marker size effects. These corrections enable investigation of long-term responses to antibiotics, which commonly can cause gradual and cumulative changes to the expression levels of a large number of genes (Lin et al., 2005; Khil and Camerini-Otero, 2002; Shaw et al., 2003) as well as evolutionary adaptations often taking place over tens of generations (Michael et al., 2016). Some of the previous studies on genome and cytosol dynamics included insights on the effects of antibiotic treatments (Weber et al., 2010a; 2012; Parry et al., 2014); however, they did not consider these corrections. In addition, these studies were limited to single time point dynamics measurements and to high (above estimated IC_50_) antibiotic doses (e.g., Weber et al., 2010a; Parry et al., 2014). Addressing these points reveals with higher precision that sub-lethal antibiotic and sorbitol treatments have small but consistent effects on genome and cytosol short timescale mobility.

### Genomic Position Affects the Degree of Response

We observe that almost all tested antibiotics (with the exception of tetracycline) cause changes to the chromosome dynamics and that these changes persist for most of the treatment time. We also show that the directions of effect are generally consistent for both Ori2 and Ter3 loci. Notably, we show that the change in the amplitude of motion depends on the initial locus mobility (before treatment). The initially fast-moving loci, Ori2, increase their mobility less when treated with ciprofloxacin and rifampicin when compared with the initially slow-moving loci, Ter3. The opposite is true for vancomycin.

The amplitude of these local motions may measure factors such as the level of genetic locus “compaction” and the macromolecular crowding of the cytosol, respectively (Weber et al., 2010a; Javer et al., 2013). Consequently, limitations to changes in motion may suggest a functional limit to maximal relaxation and compaction of a genetic locus and the ability of a bacterial cell to alter the genome's physical environment flexibly, not only depending on external stimuli but also on the chromosomal coordinate of a gene. Possible reasons for such limits include heterogeneity of the intracellular medium, of locus-specific NAP density, of locus confinement, and the physical state of the DNA molecule (torsion, elasticity, etc.).

### Deviations from “Polymer in Viscoelastic Cytosol” Model of Chromosome Dynamics Are Only Mild

Beyond the specificities, which demand future investigations, most of the responses, when plotted as fold changes in chromosomal against cytosolic mobility, lie around a straight line with slope equal to unity (Figure 3B). This general correlation in both timing and magnitude of responses between the chromosome and cytosol is overall consistent with the physical representation of the chromosome as a “polymer embedded in a viscoelastic medium” (Lampo et al., 2016; Weber et al., 2010b; Polovnikov et al., 2018). In this model, individual parts (e.g., genetic loci) of such polymer explore space by sub-diffusing through a crowded environment of macromolecules, some of which (e.g., nucleic acids and cytoskeletal filaments) possess significant elastic properties. It follows that changes to the viscosity of the surrounding medium will affect the energy states of individual polymer parts (Weber et al., 2010b; Benza et al., 2012; Bakshi et al., 2014). Consequently, we speculate that the treatment-induced chromosomal effects are a direct physical consequence of changes in the concentration of the cytosol.

We recently reported a violation of the predictions of the polymer in viscoelastic fluid model for the intracellular dynamics *immediately* after cells were compressed mechanically (Yu et al., 2018). This violation was proxied by a change in the ratio of the scaling exponents of cytoplasmic particles and chromosomal loci. Such deviation is probably due to the fact that the chromosome is not fully embedded in the cytoplasm, but is, at least in part, a separate “compartment,” consistent with the observation that the nucleoid has a different density to the cytoplasm (Valkenburg and Woldringh, 1984). A similar deviation is observed in this study (which also has slightly lower precision than the previous one), but it is quantitatively smaller (10%–16% instead of more than 20%, see Figure S3). We believe that this is likely because in this case the system has more time to equilibrate after the perturbation, as the measurements are performed more than 20 min after the perturbation. Possibly a differential role of osmotic forces from ribosomes in the presence of certain antibiotics may also play a role (Bakshi et al., 2014).

### Antibiotic-Induced Changes in Crowding Affect the Cell Globally

Should the observed chromosomal dynamics effects be a consequence of changes to the properties of the cytosol, it is the cytosolic macromolecular crowding that would mediate these effects. This would be a direct consequence of the fact that as the cytosol becomes more crowded, larger structures such as the chromosome cannot diffuse freely due to significant steric hindrance. Increased depletion-attraction interactions between crowded macromolecules may also cause the chromosome material to reduce its size for other molecules to have more space and relieve steric constraints.

Our measurements of cellular density suggest that indeed the mobility of both chromosomal and cytosolic markers is generally inversely proportional to the crowding level (Figure 5C). This finding suggests that the previously reported widespread genetic effects of antibiotics can at least partially be a consequence of antibiotics altering the nucleoprotein microenvironments of genetic loci via effects on the crowding levels.

Sophisticated mechanisms of regulation of many important cellular parameters through macromolecular crowding may be very widespread. For example, Joyner et al. (2016) have recently reported that dramatic changes to the macromolecular crowding levels in both bacteria and yeast are part of the response to glucose starvation conditions. In this context, the increase in the crowding level is thought to be a consequence of cell volume reduction and serves as a mechanism to reduce diffusional mobility and achieve homeostasis during environmental stress. More recently, Delarue et al. (2018) showed that changes in the crowding levels can be a consequence of the mTORC1 signaling pathway effects on the ribosome concentration. Using cytosolic particles of tunable size, the authors showed that macromolecule (ribosome) concentration can exert particle size-dependent effects on molecular diffusivity, opening a possibility to differentially modulate cellular reaction rates depending on particle size.

### Antibiotic-Specific Responses in Cell Concentration

Based on the aforementioned considerations, we can attempt to rationalize the antibiotic-specific effects that we have observed. For ciprofloxacin, it is plausible that the observed dynamics effects are caused primarily through inhibition of topoisomerase IV, an *E*. *coli* quinolone target secondary to DNA gyrase. Interference with decatenation of replicated DNA strands causes excessive cell filamentation and significant dilution of cytosol contents. The latter is likely to result in an increase in cytosolic aggregate and chromosomal loci mobility. Work of Weber et al. (2010a; 2012) also points to the causal role of topoisomerase IV. In their work novobiocin, an aminocoumarin antibiotic that similar to ciprofloxacin targets DNA gyrase but has a significantly lower affinity to topoisomerase IV (Hardy and Cozzarelli, 2003), caused no significant change to loci mobility.

Our rifampicin effects are generally consistent with those reported by Weber et al., who showed that after longer (≥5 but ≤30 min) treatments, chromosomal loci mobility increased and plateaued at an approximately 2-fold greater magnitude (Weber et al., 2012). Although Parry et al. (2014) reported a small decrease in the mobility of cytosolic μNS aggregates under rifampicin treatment, the tested dose was high enough (25 μg/mL, 1.5×expected MIC) to likely cause severe changes to cell physiology (e.g., near-complete shutdown of gene expression and significant growth stalling). Following Weber et al. (2012) and Bakshi et al. (2014), we propose that RNAP inhibition and mRNA pool decay, combined with subsequent ribosomal subunit-nucleoid mixing, cause a decrease in cytosol viscosity and nucleoid expansion, ultimately increasing cytosolic μNS aggregate and chromosomal loci mobility.

Interestingly, the same study reported that treatment with chloramphenicol, a protein synthesis inhibitor that (similar to tetracycline) targets the 30S ribosomal subunit to stall translation, resulted in an increase in chromosomal loci mobility. This is not consistent with our finding that tetracycline causes no consistent effect on loci mobility. However, the chloramphenicol dose used by Weber et al. (25 μg/mL, 2,500× expected MIC) was much higher than our tetracycline dose. We observed that while tetracycline was the only treatment agent that affected (and decreased) cell lengths (Figures S4 and S6 and Supplemental Information) (and also elongation rates, Figures S5 and S7 and Supplemental Information), it also increased cell widths (Figure S8 and Supplemental Information). It seems that these two changes to cell size canceled out resulting in no net change to cytosol viscosity. It is possible that responses to different doses could be different. It would also be particularly useful to establish and test a tetracycline dose that does not affect cell elongation rates as was achieved for all other treatments in this work.

For vancomycin, although we detected only very small and not consistent effects on cell lengths and elongation rates (Figures S5 and S7 and Supplemental Information), we noticed significant changes to cell morphology such as bending of cells (Figure S9 and Supplemental Information). Inhibited cell wall synthesis resulted in impaired cell elongation and thus morphological defects, possibly creating conditions for condensation of cell contents and reduced mobility of tracked markers.

For sorbitol, at least in the case of the strain harboring the marker at the Ori3 locus, our cell size measurements show a progressive volume recovery through our treatment time (Figure S5 and Supplemental Information). Examining such recovered cells with fluorescence recovery after photobleaching (FRAP), Konopka et al. observed an ∼2-fold decrease in the GFP diffusion coefficient and an ∼2-fold increase in the biopolymer volume fraction (cytoplasmic volume occupied by biopolymers such as proteins and the nucleoid) compared with pre-shock cells (Konopka et al., 2009). Our results are in general agreement with these measurements as we observed a nearly 2-fold decrease in the *MSD* of cytosolic μNS aggregates and an ∼1.3 fold increase in the macromolecular crowding level. Such changes are likely due to the intrinsic rigidity of the cell wall/outer membrane system (Rojas et al., 2018). In post-hyperosmotic shock cells, an increase in crowding is likely also due to the increased intake of osmolites during the recovery (Wood, 2006). Regarding our inability to collect valid tracks of chromosomal markers after 60–80 min of experiment, it is likely that the hyperosmotic shock conditions impaired the production of ΔParB-GFP proteins, and that some of that impairment persisted also in the recovered cells—for example, as a consequence of the increased crowding discussed earlier. Cytosolic μNS aggregates (which assembled before the shock) appeared stable throughout the experiments.

As the change in the cytosolic density is common to all proposed mechanisms and it underpins the changes to cytosolic and chromosomal dynamics, we surmise that the crowding level is the key driver of the observed dynamics. At the same time, we hypothesize that other factors—such as local differences in folded organization of the chromosome, the distributions of NAP and enzyme binding sites, etc.—contribute to the deviation from the general linear correlation between both cytosolic and chromosomal dynamics (Figure 3) and between the crowding level and marker mobility (Figure 5).

### Conclusions

By performing high-throughput and high-precision intracellular marker tracking, we discovered that sub-lethal doses of ciprofloxacin, rifampicin, and vancomycin as well as hyperosmotic shock conditions caused small but consistent changes (unique to each treatment agent) to the physical organization of chromosomal Ori2 and Ter3 loci and the cytosol. Crucially, there are strong correlations between the effects in different parts of the chromosome and between the chromosome and cytosol. Comparison of intracellular protein density under the treatments and of the magnitude of treatment-induced changes to the crowding levels lead us to the conclusion that mobility of both chromosomal and cytosolic markers is generally inversely proportional to the crowding level. We conclude that antibiotics, by affecting the macromolecular composition of the cell, can alter the physical microenvironments of the genome and the biosynthetic machinery, potentially affecting expression levels of a large number of genes.

Based on our findings, we propose specific mechanisms—consistent with known modes of action and with the current physical view of the bacterial chromosome and cytoplasm—on how different antibiotics can exert their effects. In brief, our results show that the main physical chromosomal and cytosolic responses to a wide range of sub-lethal treatments can be interpreted in the context of intracellular crowding. In this framework, we speculate that these mechanisms could contribute to switch, or tip, crowding homeostasis (van den Berg et al., 2017), in a way that is qualitatively generic to any antibiotic treatment or stress, but quantitatively specific to each perturbation. Crowding could itself affect growth (Klumpp and Hwa, 2014; Scott et al., 2014), thus affecting fitness changes even at mild levels of perturbations. We are just beginning to reveal this interplay between physical degrees of freedom and the global state of a cell, and indeed there is one experiment in our study, the motilities after perturbation with vancomycin, which is not explainable as a simple consequence of crowding.

### Limitations of the Study

Our work portrays changes in intracellular motility and molecular crowding due to antibiotic treatment, but does not delve on the mechanisms through which these come to be. Our measurements of cellular density were carried out at the population level, therefore requiring a certain amount of handling of the samples, which could introduce experimental errors. Although we tried to exclude possible sources of artifactual nature with our controls, investigations of crowding at the single-cell level, which we could not pursue due to a lack of tools, would be an interesting way to confirm our population-level findings, particularly for the cases of rifampicin and vancomycin treatments in which biomass to OD changes are smaller.

### Resource Availability

#### Lead Contact

Pietro Cicuta pc245@cam.ac.uk.

#### Materials Availability

Further information including reasonable requests for materials should be directed to and will be fulfilled by the Lead Contact, pc245@cam.ac.uk.

#### Data and Code Availability

The code is available from https://github.com/ver228/bacteria-loci-tracker and data are available from 10.5281/zenodo.3836129.

## Methods

All methods can be found in the accompanying Transparent Methods supplemental file.

## Supplemental Information

Document S1. Transparent Methods, Figures S1–S13, and Tables S1–S3

## Footnotes

Supplemental Information can be found online at https://doi.org/10.1016/j.isci.2020.101560.

## References

[bib1] Bakshi S., Choi H., Mondal J., Weisshaar J.C. (2014). Time-dependent effects of transcription- and translation-halting drugs on the spatial distributions of the E. coli chromosome and ribosomes. Mol. Microbiol..

[bib2] Barer R., Tkaczyk S. (1954). Refractive index of concentrated protein solutions. Nature.

[bib3] Basan M., Hui S., Okano H., Zhang Z., Shen Y., Williamson J.R., Hwa T. (2015). Overflow metabolism in escherichia coli results from efficient proteome allocation. Nature.

[bib4] Basan M., Zhu M., Dai X., Warren M., Sévin D., Wang Y.P., Hwa T. (2015). Inflating bacterial cells by increased protein synthesis. Mol. Sys. Biol..

[bib5] Bateman J.B., Wagman J., Carstensen E.L. (1966). Refraction and absorption of light in bacterial suspensions. Kolloid-Zeitschrift Z. für Polymere.

[bib6] Benza V.G., Bassetti B., Dorfman K.D., Scolari V.F., Bromek K., Cicuta P., Lagomarsino M.C. (2012). Physical descriptions of the bacterial nucleoid at large scales, and their biological implications. Rep. Prog. Phys..

[bib7] van den Berg J., Boersma A.J., Poolman B. (2017). Microorganisms maintain crowding homeostasis. Nat. Rev. Microbiol..

[bib8] Bollenbach T., Quan S., Chait R., Kishony R. (2009). Nonoptimal microbial response to antibiotics underlies Suppressive drug interactions. Cell.

[bib9] Bremer H., Dennis P.P. (2008). Modulation of chemical composition and other parameters of the cell at different exponential growth rates. EcoSal Plus.

[bib10] Brown M.R., Collier P.J., Gilbert P. (1990). Influence of growth rate on susceptibility to antimicrobial agents: modification of the cell envelope and batch and continuous culture studies. Antimicrob. Agents Chemother..

[bib11] Cass J.A., Kuwada N.J., Traxler B., Wiggins P.A. (2016). Escherichia coli chromosomal loci segregate from midcell with universal dynamics. Biophys. J..

[bib12] Cicuta P., Donald A.M. (2007). Microrheology: a review of the method and applications. Soft Matter.

[bib13] Crespi A., Lobino M., Matthews J.C.F., Politi A., Neal C.R., Ramponi R., Osellame R., O’Brien J.L. (2012). Measuring protein concentration with entangled photons. Appl. Phys. Lett..

[bib14] Crozat E., Tardin C., Salhi M., Rousseau P., Lablaine A., Bertoni T., Holcman D., Sclavi B., Cicuta P., Cornet F. (2019). Multiple activities of the matp protein are involved in post-replicative pairing of sister chromosomes in escherichia coli. bioRxiv.

[bib15] Dalebroux Z.D., Swanson M.S. (2012). ppGpp: magic beyond RNA polymerase. Nat. Rev. Microbiol..

[bib16] Dame R.T. (2005). The role of nucleoid-associated proteins in the organization and compaction of bacterial chromatin. Mol. Microbiol..

[bib17] Dame R.T., Kalmykowa O.J., Grainger D.C. (2011). Chromosomal macrodomains and associated proteins: implications for DNA organization and replication in gram negative bacteria. PLoS Genet..

[bib18] Delarue M., Brittingham G.P., Pfeffer S., Surovtsev I.V., Pinglay S., Kennedy K.J., Schaffer M., Gutierrez J.I., Sang D., Poterewicz G. (2018). mtorc1 controls phase separation and the biophysical properties of the cytoplasm by tuning crowding. Cell.

[bib19] Espéli O., Borne R., Dupaigne P., Thiel A., Gigant E., Mercier R., Boccard F. (2012). A MatP-divisome interaction coordinates chromosome segregation with cell division in E. coli. EMBO J..

[bib20] Espeli O., Mercier R., Boccard F. (2008). DNA dynamics vary according to macrodomain topography in the E. coli chromosome. Mol. Microbiol..

[bib21] Folsom J.P., Parker A.E., Carlson R.P. (2014). Physiological and proteomic analysis of escherichia coli iron-limited chemostat growth. J. Bacteriol..

[bib22] Greco W.R., Bravo G., Parsons J.C. (1995). The search for synergy: a critical review from a response surface perespective. Phar. Rev..

[bib23] Greulich P., Scott M., Evans M.R., Allen R.J. (2015). Growth-dependent bacterial susceptibility to ribosome-targeting antibiotics. Mol. Syst. Biol..

[bib24] Gupta R.S. (2011). Origin of diderm (Gram-negative) bacteria: antibiotic selection pressure rather than endosymbiosis likely led to the evolution of bacterial cells with two membranes. Antonie van Leeuwenhoek.

[bib25] Hardy C.D., Cozzarelli N.R. (2003). Alteration of Escherichia coli topoisomerase IV to novobiocin resistance. Antimicrob. Agents Chemother..

[bib26] Hoffman L.R., D’Argenio D.A., MacCoss M.J., Zhang Z., Jones R.A., Miller S.I. (2005). Aminoglycoside antibiotics induce bacterial biofilm formation. Nature.

[bib27] Javer A., Kuwada N.J., Long Z., Benza V.G., Dorfman K.D., Wiggins P.a., Cicuta P., Lagomarsino M.C. (2014). Persistent super-diffusive motion of Escherichia coli chromosomal loci. Nat. Commun..

[bib28] Javer A., Long Z., Nugent E., Grisi M., Siriwatwetchakul K., Dorfman K.D., Cicuta P., Cosentino Lagomarsino M. (2013). Short-time movement of E. coli chromosomal loci depends on coordinate and subcellular localization. Nat. Commun..

[bib29] Jerman B., Butala M., Zgur-Bertok D. (2005). Sublethal concentrations of ciprofloxacin induce bacteriocin synthesis in Escherichia coli. Antimicrob. Agents Chemother..

[bib30] Joyner R.P., Tang J.H., Helenius J., Dultz E., Brune C., Holt L.J., Huet S., Müller D.J., Weis K. (2016). A glucose-starvation response regulates the diffusion of macromolecules. Elife.

[bib31] Khil P.P., Camerini-Otero R.D. (2002). Over 1000 genes are involved in the DNA damage response of Escherichia coli. Mol. Microbiol..

[bib32] Klumpp S., Hwa T. (2008). Growth-rate-dependent partitioning of RNA polymerases in bacteria. Proc. Natl. Acad. Sci. U S A.

[bib33] Klumpp S., Hwa T. (2014). Bacterial growth: global effects on gene expression, growth feedback and proteome partition. Curr. Opin. Biotechnol..

[bib34] Konopka M.C., Shkel I.A., Cayley S., Record M.T., Weisshaar J.C. (2006). Crowding and confinement effects on protein diffusion in vivo. J. Bacteriol..

[bib35] Konopka M.C., Sochacki K.A., Bratton B.P., Shkel I.A., Record M.T., Weisshaar J.C. (2009). Cytoplasmic protein mobility in osmotically stressed Escherichia coli. J. Bacteriol..

[bib36] Kwon Y.K., Higgins M.B., Rabinowitz J.D. (2010). Antifolate-induced depletion of intracellular glycine and purines inhibits thymineless death in E. coli. ACS Chem. Biol..

[bib37] Lampo T.J., Kennard A.S., Spakowitz A.J. (2016). Physical modeling of dynamic coupling between chromosomal loci. Biophys. J..

[bib38] Lin J.T., Connelly M.B., Amolo C., Yaver D.S., Otani S. (2005). Global transcriptional response of bacillus subtilis to treatment with subinhibitory concentrations of antibiotics that inhibit protein synthesis. Antimicrob. Agents Chemother..

[bib39] Long C.P., Gonzalez J.E., Sandoval N.R., Antoniewicz M.R. (2016). Characterization of physiological responses to 22 gene knockouts in escherichia coli central carbon metabolism. Metab. Eng..

[bib40] Marquis R.E. (1973). Immersion refractometry of isolated bacterial cell walls. J. Bacteriol..

[bib41] Michael B., Lieberman T.D., Kelsic E.D., Chait R., Gross R., Yelin I., Kishony R. (2016). Spatiotemporal microbial evolution on antibiotic landscapes. Science.

[bib42] Miermont A., Waharte F., Hu S., McClean M., Bottani S., Léon S., Hersen P. (2013). Severe osmotic compression triggers a slowdown of intracellular signaling, which can be explained by molecular crowding. Proc. Natl. Acad. Sci. U S A.

[bib43] Mitosch K., Bollenbach T. (2014). Bacterial responses to antibiotics and their combinations. Environ. Microbiol. Rep..

[bib44] Oldewurtel E.R., Kitahara Y., Cordier B., Özbaykal G., van Teeffelen S. (2019). Bacteria control cell volume by coupling cell-surface expansion to dry-mass growth. bioRxiv.

[bib45] Parry B.R., Surovtsev I.V., Cabeen M.T., O’Hern C.S., Dufresne E.R., Jacobs-Wagner C. (2014). The bacterial cytoplasm has glass-like properties and is fluidized by metabolic activity. Cell.

[bib46] Pelletier J., Halvorsen K., Ha B.Y., Paparcone R., Sandler S., Woldringh C., Wong W., Jun S. (2012). Physical manipulation of the escherichia coli chromosome reveals its soft nature. Proc. Natl. Acad. Sci. U S A.

[bib47] Polovnikov K.E., Gherardi M., Cosentino-Lagomarsino M., Tamm M.V. (2018). Fractal folding and medium viscoelasticity contribute jointly to chromosome dynamics. Phys. Rev. Lett..

[bib48] Rahal J.J., Simberkoff M.S. (1979). Bactericidal and bacteriostatic action of chloramphenicol against meningeal pathogens. Antimicrob. Agents Chemother..

[bib49] Ren Q., Henes B., Fairhead M., Thöny-Meyer L. (2013). High level production of tyrosinase in recombinant escherichia coli. BMC Biotechnol..

[bib50] Rojas E., Theriot J.A., Huang K.C. (2014). Response of Escherichia coli growth rate to osmotic shock. Proc. Natl. Acad. Sci. U S A.

[bib51] Rojas E.R., Billings G., Odermatt P.D., Auer G.K., Zhu L., Miguel A., Chang F., Weibel D.B., Theriot J.A., Huang K.C. (2018). The outer membrane is an essential load-bearing element in gram-negative bacteria. Nature.

[bib52] Scott M., Gunderson C.W., Mateescu E.M., Zhang Z., Hwa T. (2010). Interdependence of cell growth and gene expression: origins and consequences. Science.

[bib53] Scott M., Klumpp S., Mateescu E.M., Hwa T. (2014). Emergence of robust growth laws from optimal regulation of ribosome synthesis. Mol. Syst. Biol..

[bib54] Shaw K.J., Miller N., Liu X., Lerner D., Wam J., Bittner A., Morrow B. (2003). Comparison of the changes in global gene expression of Escherichia coli induced by four bactericidal agents. J. Mol. Microbiol. Biotechnol..

[bib55] Sobetzko P., Travers A., Muskhelishvili G. (2012). Gene order and chromosome dynamics coordinate spatiotemporal gene expression during the bacterial growth cycle. Proc. Natl. Acad. Sci. U S A.

[bib56] Stavans J., Oppenheim A. (2006). DNA-protein interactions and bacterial chromosome architecture. Phys. Biol..

[bib57] Stevenson K., McVey A.F., Clark I.B.N., Swain P.S., Pilizota T. (2016). General calibration of microbial growth in microplate readers. Sci. Rep..

[bib58] Tsai H., Nelliat A., Choudhury M., Kucharavy A., Bradford W.D., Cook M.E., Kim J., Mair D.B., Sun S.X., Schatz M.C., Li R. (2019). Hypo-osmotic-like stress underlies general cellular defects of aneuploidy. Nature.

[bib59] Tuomanen E., Cozens R., Tosch W., Zak O., Tomasz A. (1986). The rate of killing of Escherichia coli by beta-lactam antibiotics is strictly proportional to the rate of bacterial growth. J. Gen. Microbiol..

[bib60] Valkenburg J., Woldringh C. (1984). Phase separation between nucleoid and cytoplasm in *Escherichia coli* as defined by immersive refractometry. J. Bacteriol..

[bib61] Waigh T.A. (2005). Microrheology of complex fluids. Rep. Prog. Phys..

[bib62] Weber S.C., Spakowitz A.J., Theriot J.A. (2010). Bacterial chromosomal loci move subdiffusively through a viscoelastic cytoplasm. Phys. Rev. Lett..

[bib63] Weber S.C., Theriot J.A., Spakowitz A.J. (2010). Subdiffusive motion of a polymer composed of subdiffusive monomers. Phys. Rev. E Stat. Nonlin Soft Matter Phys..

[bib64] Weber S.C., Spakowitz A.J., Theriot J.A. (2012). Nonthermal ATP-dependent fluctuations contribute to the in vivo motion of chromosomal loci. Proc. Natl. Acad. Sci. U S A.

[bib65] Wiegand I., Hilpert K., Hancock R.E.W. (2008). Agar and broth dilution methods to determine the minimal inhibitory concentration (MIC) of antimicrobial substances. Nat. Protoc..

[bib66] Wlodarski M., Raciti B., Kotar J., Cosentino Lagomarsino M., Fraser G.M., Cicuta P. (2017). Both genome and cytosol dynamics change in E. coli challenged with sublethal rifampicin. Phys. Biol..

[bib67] Wood J.M. (2006). Osmosensing by bacteria. Sci. Signal..

[bib68] Wood T.K., Knabel S.J., Kwan B.W. (2013). Bacterial persister cell formation and dormancy. Appl. Environ. Microbiol..

[bib69] You C., Okano H., Hui S., Zhang Z., Kim M., Gunderson C.W., Wang Y.P., Lenz P., Yan D., Hwa T. (2013). Coordination of bacterial proteome with metabolism by cyclic amp signalling. Nature.

[bib70] Yu S., Sheats J., Cicuta P., Sclavi B., Cosentino Lagomarsino M., Dorfman K.D. (2018). Subdiffusion of loci and cytoplasmic particles are different in compressed cells. Commun. Biol..

