# Supplementary material for: Cytosolic Crowding Drives the Dynamics of Both Genome and Cytosol in Escherichia coli Challenged with Sub-lethal Antibiotic Treatments
Source: iScience. 2020 Sep 15;23(10):101560. doi: 10.1016/j.isci.2020.101560 (PMC7522891; doi:10.1016/j.isci.2020.101560)
Supplement: Document S1. Transparent Methods, Figures S1–S13, and Tables S1–S3 [file mmc1.pdf]

iScience, Volume *23*

## Supplemental Information

# Cytosolic Crowding Drives the Dynamics of Both Genome and Cytosol in *Escherichia coli* Challenged with Sub-lethal Antibiotic Treatments

**Michal Wlodarski, Leonardo Mancini, Bianca Raciti, Bianca Sclavi, Marco Cosentino Lagomarsino, and Pietro Cicuta**

# Supplementary materials of: Cytosolic crowding drives the dynamics of both genome and cytosol in *Escherichia coli* challenged with sublethal antibiotic treatments

Michal Wlodarski*

*Biological and Soft Systems, Cavendish Laboratory, University of Cambridge, United Kingdom*

Leonardo Mancini*

*Biological and Soft Systems, Cavendish Laboratory, University of Cambridge, United Kingdom*

Bianca Raciti

*Biological and Soft Systems, Cavendish Laboratory, University of Cambridge, United Kingdom*

Bianca Sclavi

*Laboratory of Biology and Applied Pharmacology (UMR 8113 CNRS), École Normale Supérieure, Paris-Saclay, France*

Marco Cosentino Lagomarsino**

*Laboratory of Computational and Quantitative Biology (UMR 7238 CNRS), Sorbonne Université, Paris, France;*

Pietro Cicuta**

*Biological and Soft Systems, Cavendish Laboratory, University of Cambridge, United Kingdom*

[a] *IFOM Foundation FIRC Institute of Molecular Oncology Foundation, Milan 20139, Italy;*
[b] *Dipartimento di Fisica and I.N.F.N., Università degli Studi di Milano, Via Celoria 16, I-20133 Milano, Italy.*

---

---

*Equal contribution
**Corresponding author
*Email addresses:* `marco.cosentino-lagomarsino@ifom.eu` (Marco Cosentino Lagomarsino), `pc245@cam.ac.uk` (Pietro Cicuta)

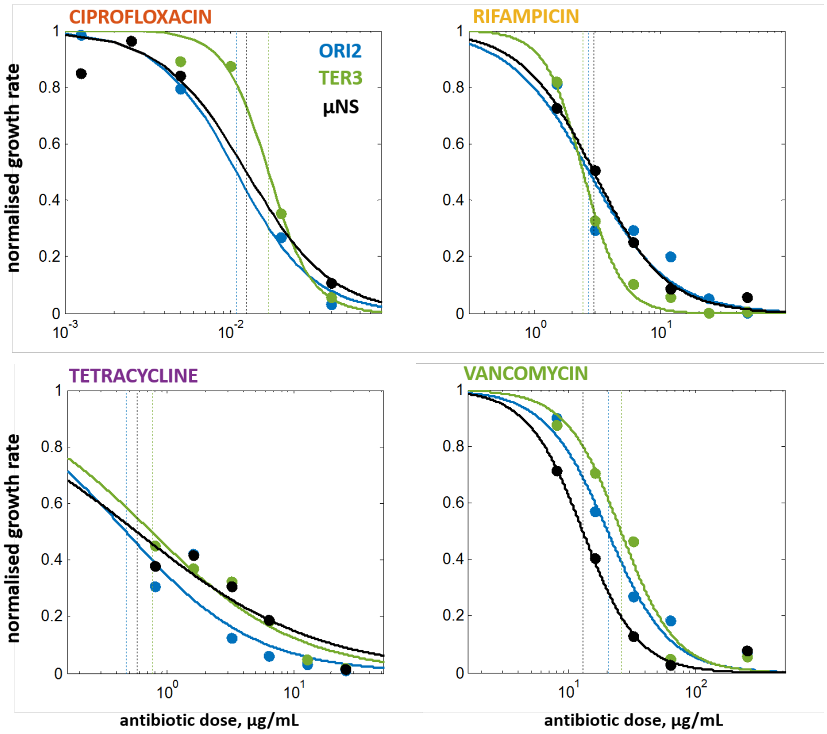


Figure S1: **Dose-response curves used for the $IC_{50}$ concentrations determination, related to Figure 1.** Slopes of the linear parts of the growth curves obtained at varying concentrations of antibiotics were calculated and the means were used to construct the dose-response curves. After fitting with Equation S1, $IC_{50}$ concentrations (*dashed vertical lines*) were selected experiments (Table S3). Curve colours represent tested strains: Ori2 (*blue*), Ter3 (*green*), and $\mu$NS (*black*).

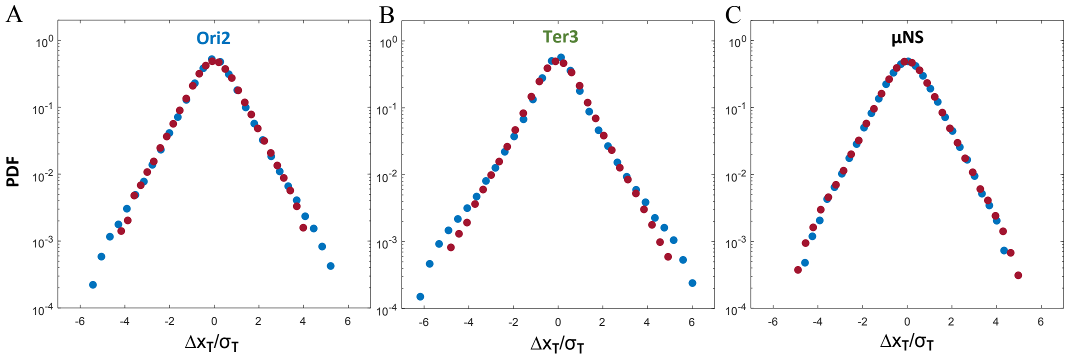


Figure S2: **Step-size distributions for $\tau = 0.1$ s after 1 h treatment with sorbitol, related to Figure 2.** Panels show normalised probability distributions of step sizes at $\tau$=0.1 s along a fixed $x$-direction normalised by standard deviation for **(A)** Ori2 and **(B)** Ter3 chromosomal loci, and **(C)** cytosolic $\mu$NS aggregates, tracked on agar. Data is binned into an arbitrary number of 32 linearly spaced out bins; bins containing at least 50 steps are shown. No significant difference between the control and sorbitol-treated data was observed.

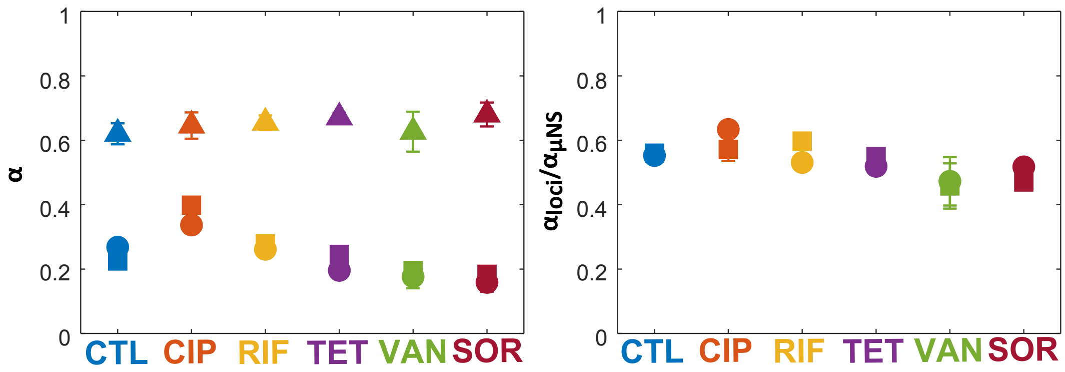


Figure S3: **Some of the treatment conditions change the scaling exponent $\alpha$, related to Figure 2.** The *left* panel shows the scaling exponent $\alpha$ for all tested treatments at $T_{treat} = 20$ min (Ori2, *circles*; Ter3, *squares*; $\mu$NS, *triangles*). Error bars show the standard deviations of the medians of the distributions divided by the square root of the number of biological replicates ($n =$ 9 and 6 for chromosomal and cytosolic markers, respectively); in most cases the error bars are smaller than the plot markers. The *right* panel shows the loci/$\mu$NS ratios of the scaling exponents (Ori2/$\mu$NS, *circles*; Ter3/$\mu$NS, *squares*. The error bars represent propagated standard deviations. The antibiotic-induced changes to $\alpha$ observed in this work are much smaller (change of up to 16% at the shortest $T_{treat}$) compared those in compressed cells (change of at least 20% at 20 psi for all markers, as reported previously by Shi et al. (Yu et al., 2018)).

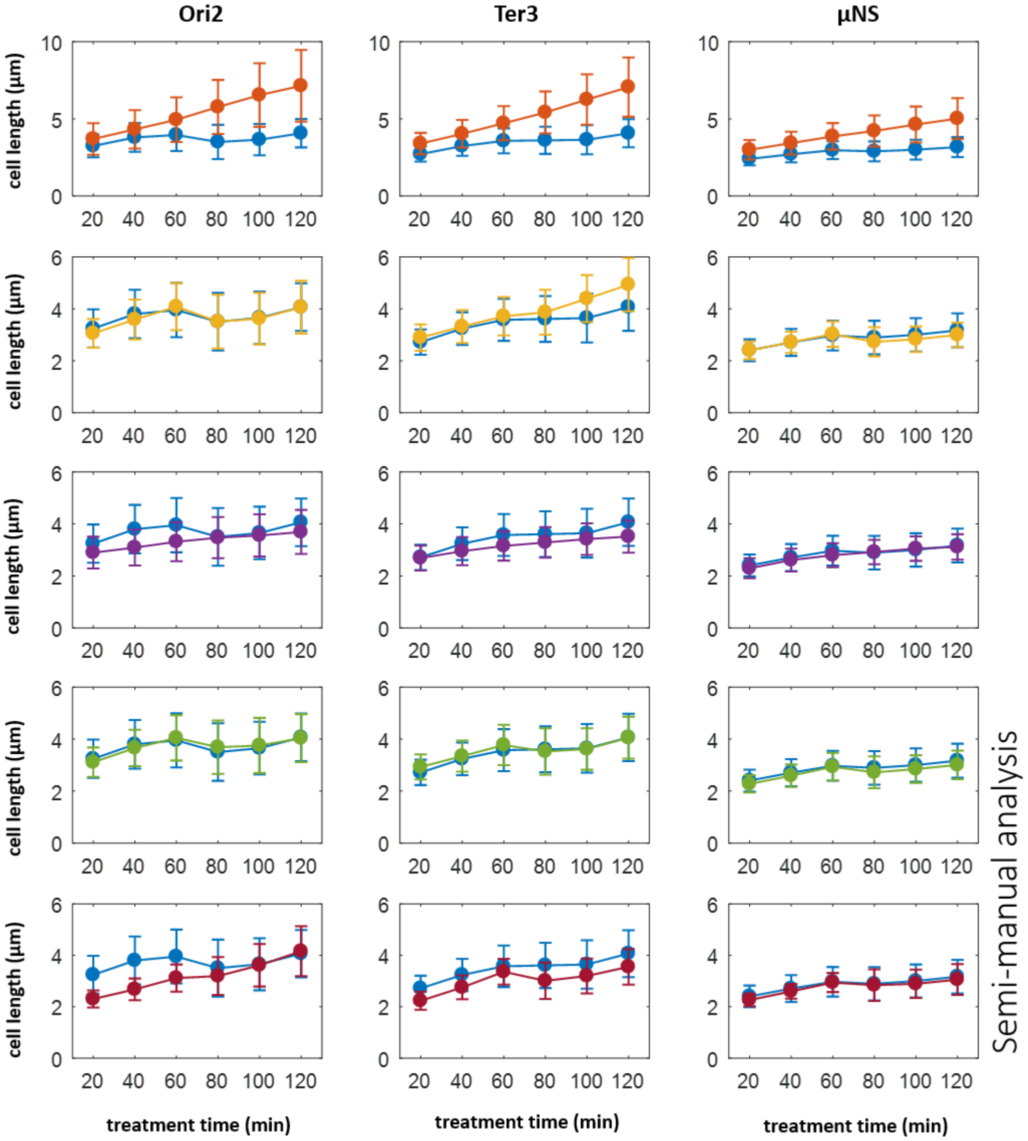


Figure S4: **Cell lengths measured semi-manually, related to Figure 5.** Strains are indicated at the top of each column of plots; treated with ciprofloxacin (*orange*), rifampicin (*yellow*), tetracycline (*violet*), vancomycin (*green*), and sorbitol (*red*); and the controls (*blue*).

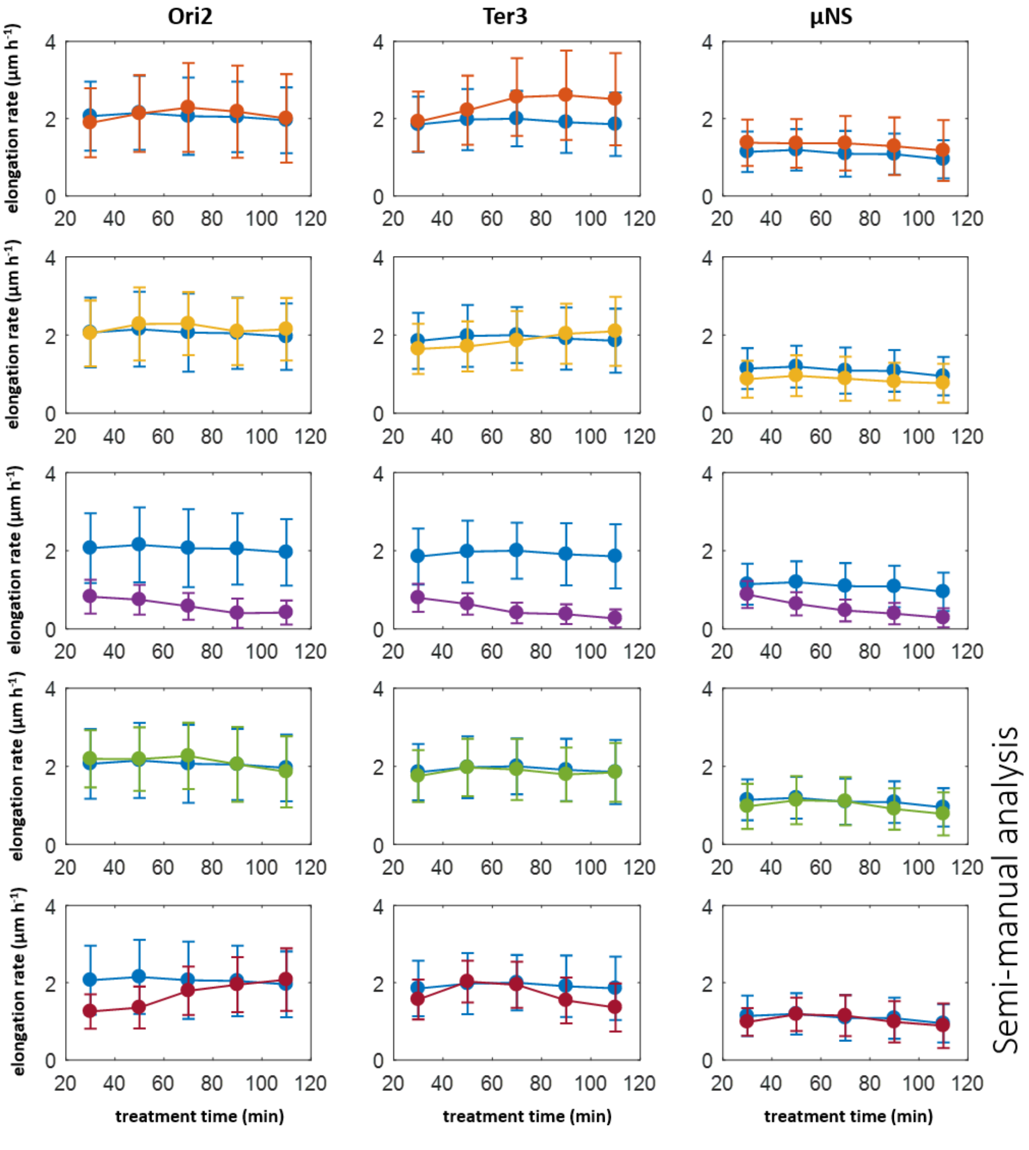


Figure S5: **Cell elongation rates measured semi-manually, related to Figure 5.** Strains are indicated at the top of each column of plots; treated with ciprofloxacin (*orange*), rifampicin (*yellow*), tetracycline (*violet*), vancomycin (*green*), and sorbitol (*red*); and the controls (*blue*).

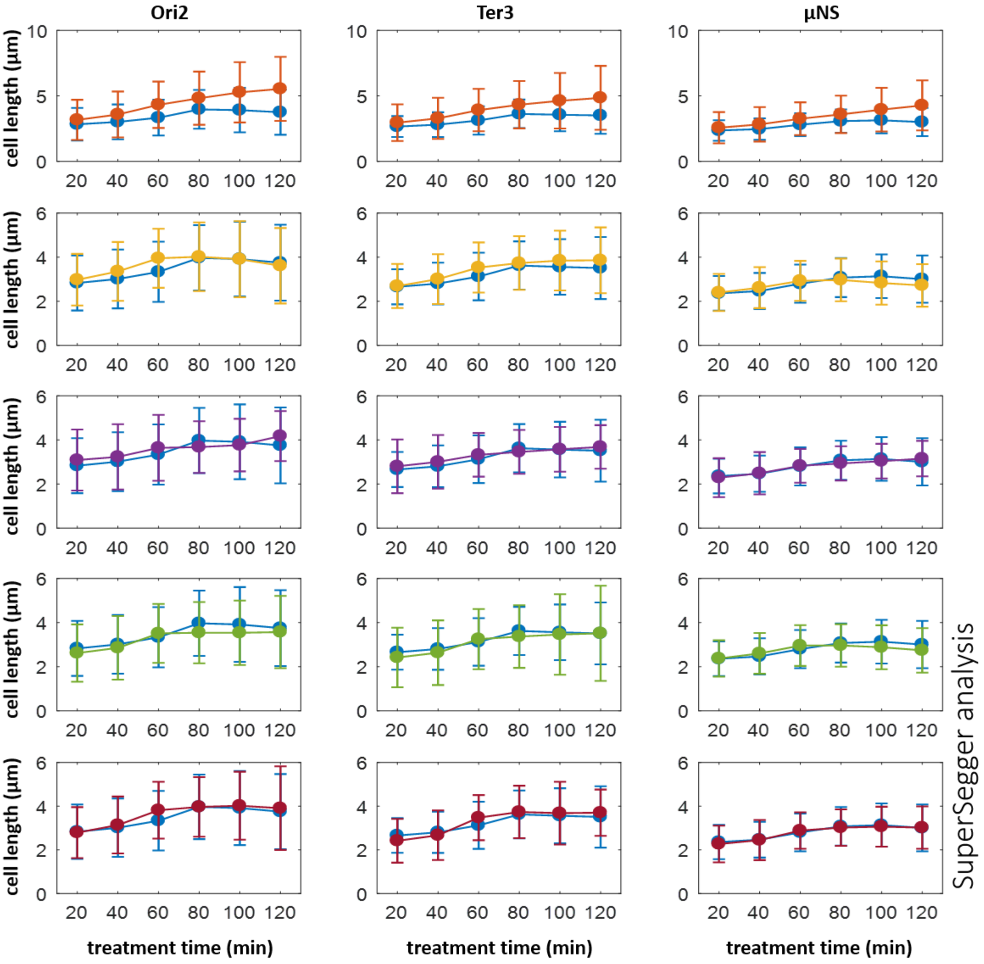


Figure S6: **Cell lengths measured with SuperSegger software, related to Figure 5.** Strains are indicated at the top of each column of plots; treated with ciprofloxacin (*orange*), rifampicin (*yellow*), tetracycline (*violet*), vancomycin (*green*), and sorbitol (*red*); and the controls (*blue*).

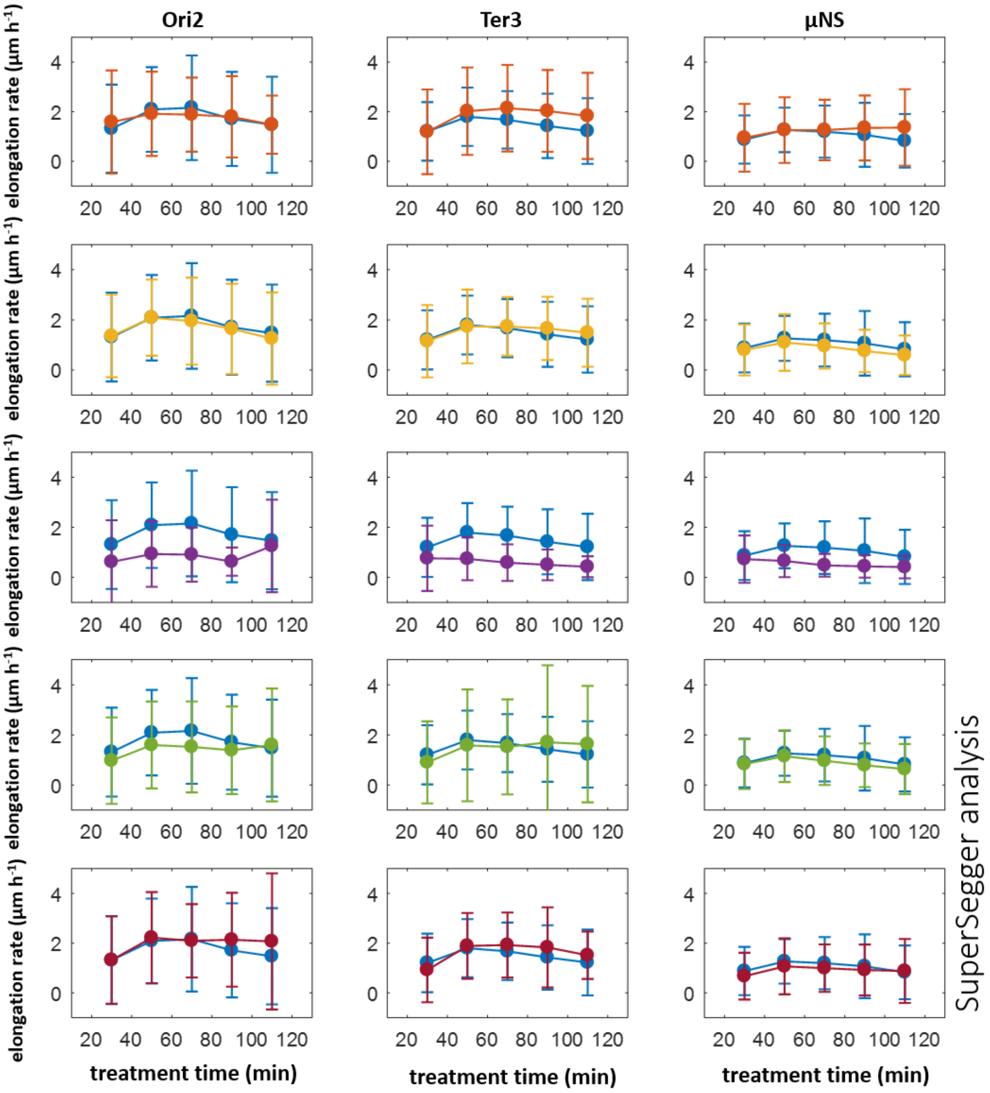


Figure S7: **Cell elongation rates measured with SuperSegger software, related to Figure 5.** Strains are indicated at the top of each column of plots; treated with ciprofloxacin (*orange*), rifampicin (*yellow*), tetracycline (*violet*), vancomycin (*green*), and sorbitol (*red*); and the controls (*blue*).

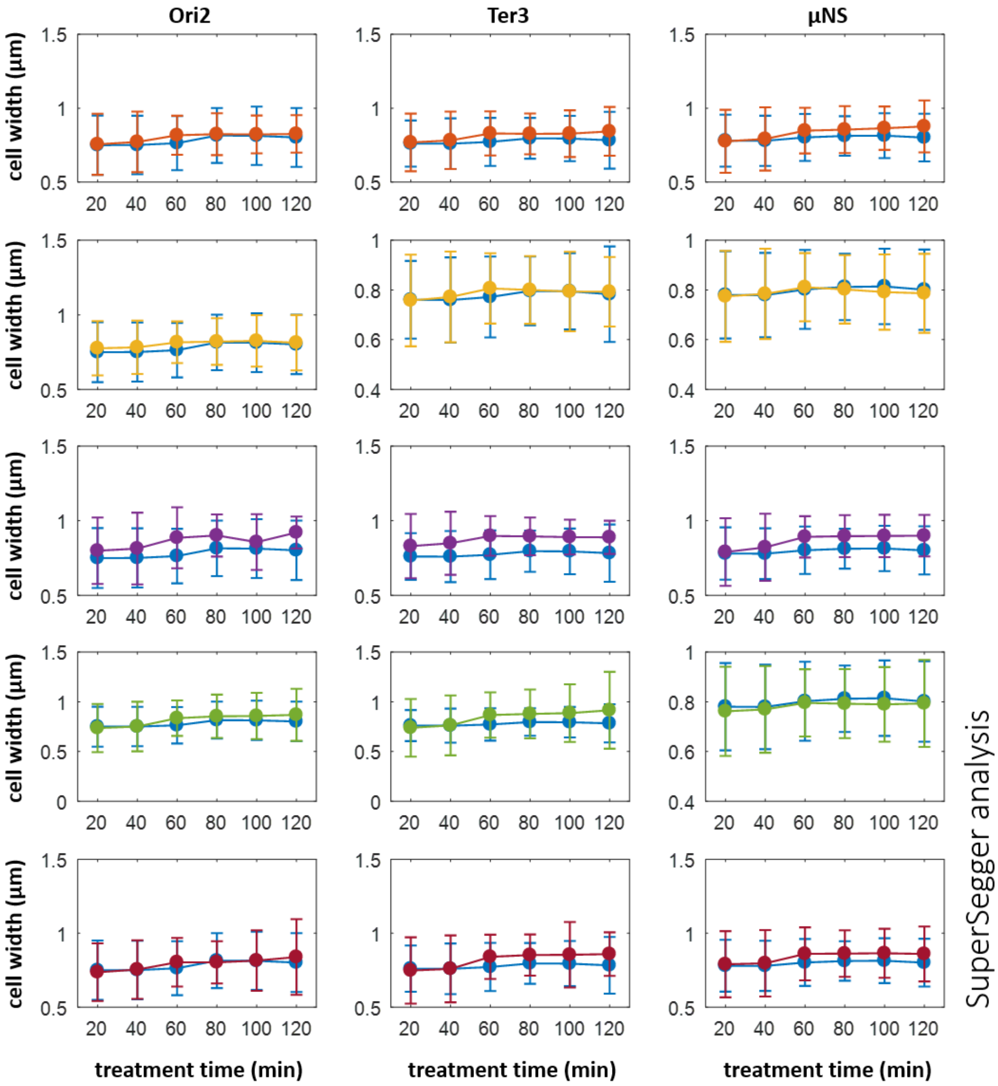


Figure S8: **Cell widths measured with SuperSegger software, related to Figure 5.** Strains are indicated at the top of each column of plots; treated with ciprofloxacin (*orange*), rifampicin (*yellow*), tetracycline (*violet*), vancomycin (*green*), and sorbitol (*red*); and the controls (*blue*).

| | experiment # | bio. repl. | control | ciprofloxacin | rifampicin | tetracycline | vancomycin | sorbitol | |
|---|---|---|---|---|---|---|---|---|---|
| | 1. | 3 | 7,985 | 5,015 | 1,986 | 451 | 2,608 | 1,356 | Ori2 |
| | 2. | 3 | 4,051 | 2,556 | 3,443 | 712 | 2,110 | 338 | |
| | 3. | 3 | 1,728 | 1,977 | 2,528 | 2,768 | 1,356 | 2,001 | |
| *subtotals* | *3* | *9* | *13,764* | *9,548* | *7,957* | *3,931* | *6,074* | *3,695* | |
| | 4. | 3 | 1,456 | 3,387 | 4,718 | 1,440 | 1,342 | 868 | Ter3 |
| | 5. | 3 | 3,214 | 3,199 | 3,499 | 2,560 | 2,698 | 454 | |
| | 6. | 3 | 1,694 | 1,813 | 2,649 | 1,243 | 3,601 | 888 | |
| *subtotals* | *3* | *9* | *6,364* | *8,399* | *10,866* | *5,243* | *7,641* | *2,210* | |
| | 7. | 3 | 1,230 | 841 | 784 | 10,66 | 746 | 1,837 | GFP-µNS |
| | 8. | 3 | 760 | 743 | 1,127 | 298 | 1,078 | 948 | |
| *subtotals* | *2* | *6* | *1,990* | *1,584* | *1,911* | *1,364* | *1,824* | *2,785* | |
| *TOTALS* | *8* | *24* | *22,118* | *19,531* | *20,734* | *10,538* | *15,539* | *8,690* | *97,150* |

Table S1: **Number of collected and analysed tracks and biological replicates for individual experiments, related to Figure 1.** Numbers represent sums of tracks acquired at all measurement time points (6 every 20 min). For each marker type, three experiments – each for 3 (chromosomal loci) or 2 (cytosolic aggregates) biological replicates (refer to Section 1.2 of Methods for details) were completed. In total, at least 1,364 ($\mu$NS, tetracycline) and up to 13,764 (Ori2, control) tracks were collected per treatment condition. The total of 97,150 tracks were collected and analysed for entire work.

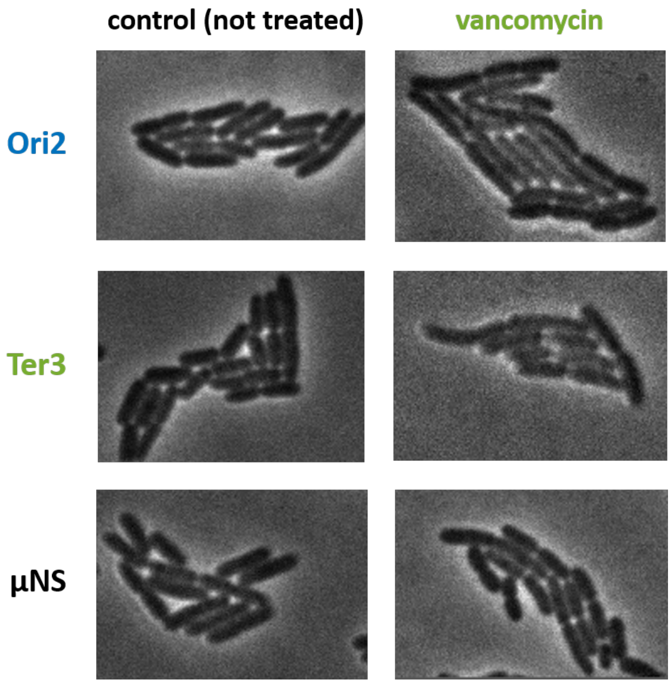


Figure S9: **Vancomycin changes cell morphology, related to Figure 5.** Example phase contrast images of three *E. coli* strains under no treatment and sub-lethal vancomycin. Treatment with vancomycin affects cell shape causing unusual bending of cells.

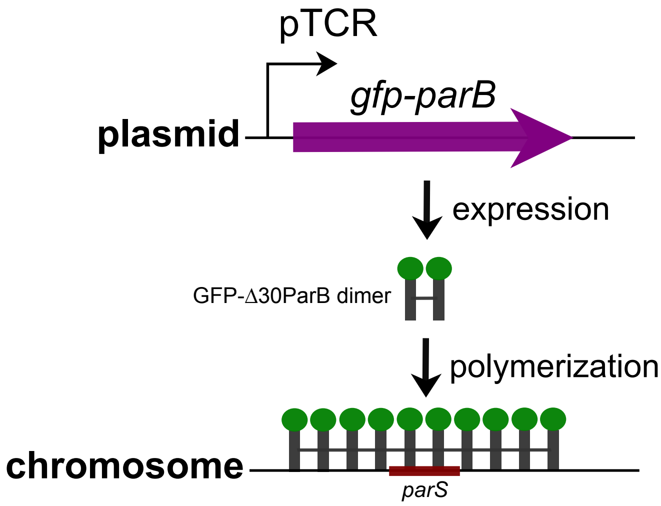


Figure S10: **Schematic representation of the ParB-*parS* chromosomal loci labelling system, related to Figure 1.** Expression of the plasmid-based fluorescent *gfp-parB* fusion protein is under control of an ITPG-inducible pTCR promoter. The truncated Δ30 ParB protein fused to GFP forms a dimer in solution but polymerases when bound to the chromosomal *parS* binding site, forming a trackable fluorescent marker.

| Treatment | Ori2 p-value | Ter3 p-value | Cyt p-value |
|---|---|---|---|
| Ciprofloxacin | 0.003 | $<0.001$ | $<0.001$ |
| Rifampicin | 0.614 | 0.028 | 0.168 |
| Tetracycline | 0.619 | 0.025 | 0.292 |
| Vancomycin | $<0.001$ | 0.016 | 0.048 |
| Sorbitol | $<0.001$ | $<0.001$ | $<0.001$ |

Table S2: **Statistical significance of the changes in MSD shown in Fig. 1 of the main text, related to Figure 1.** The MSD of the markers shown remains overall constant over treatment time as shown in Figure 1 such that trends can be fitted by straight lines parallel to the $x$ axis, with a constant $y$ value equal to the mean of the data points over time. Because every data point is in turn the result of the average of the medians of different biological replicates, each median was considered representative of MSD and the statistical significance of their changes assayed with a t-test. P-values are given.

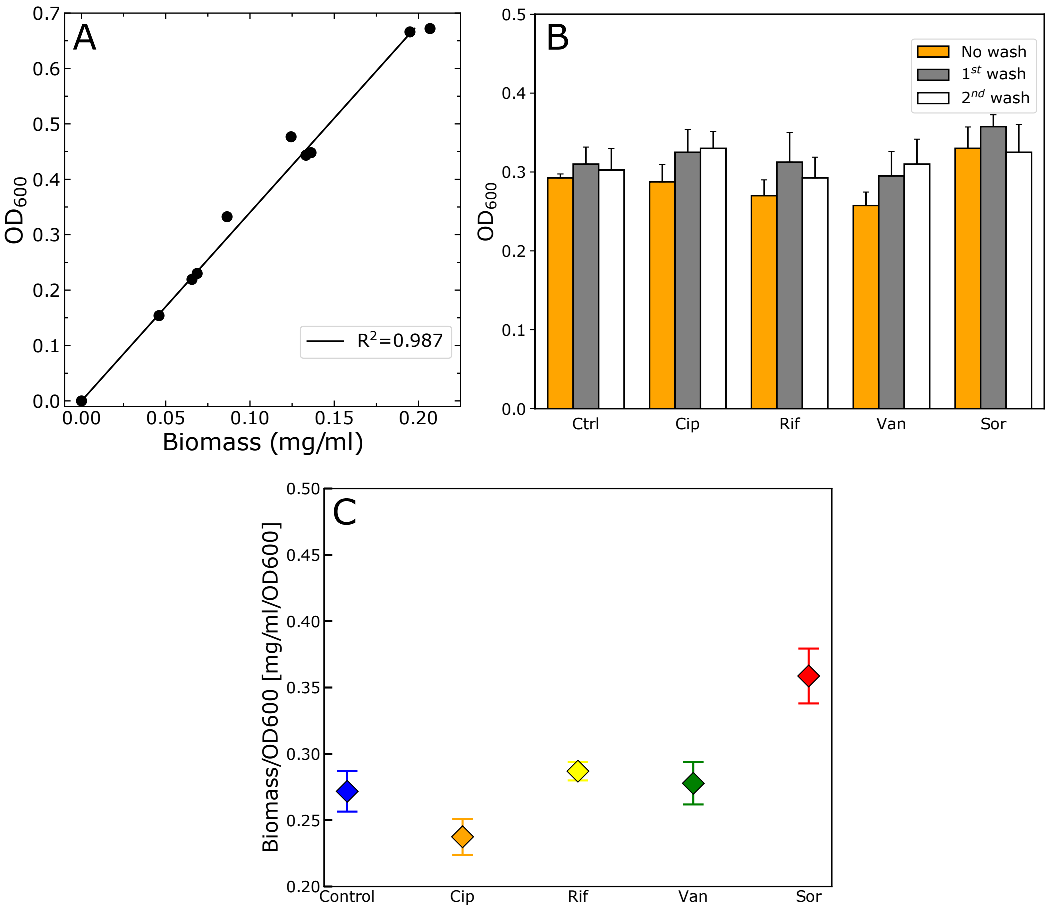


Figure S11: **Measurements of optical density and dry mass enable the estimation of cell density variations, related to Figure 5.** (**A**) $OD_{600}$ to dry mass linear correlation for untreated cells. Results from 3 biological replicates. To ensure that the correlation was assayed for cells with the same properties, each sample was collected and used to produce 3 dilutions: 1:0, 2:3 and 1:3. (**B**) Washing of the samples via centrifugation and resuspension in water does not cause differential cell lysis. Optical density of unwashed cells (orange), cells washed once in water (grey) and cells washed twice in water (white) are given. Bars are the average of 4 replicates and the error bars show the standard deviation. (**C**) Experiments presented in Figure 6A of the main text were repeated on ice to exclude artifacts due to cell growth during sample handling. Data points show the average of 3 replicates with the error bars showing the standard deviation.

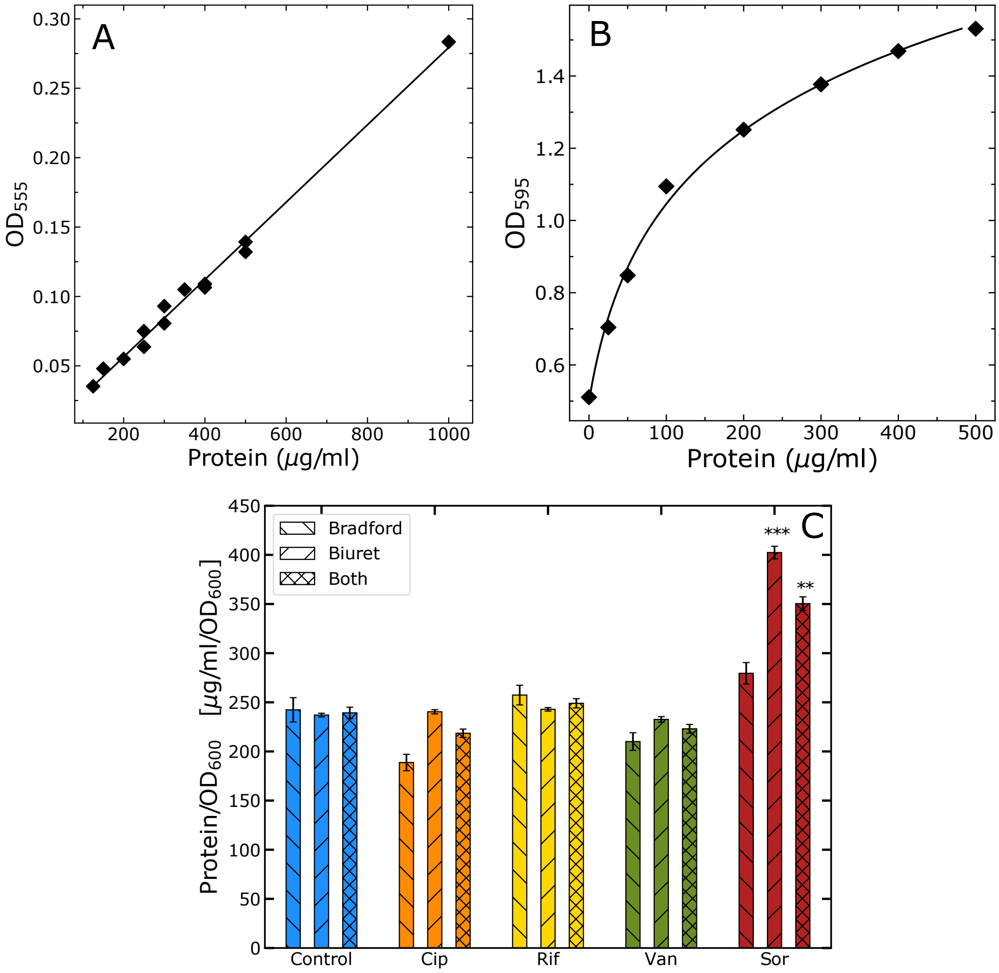


Figure S12: **Comparison of protein estimation results from the Biuret and Bradford assays, related to Figure 5.** (**A**) Biuret assay calibration curve with BSA protein. Data was fitted to a straight line: $aProtein + b$ where a = 0.00028 and b = 0.0007 are fitting parameters. (**B**) Bradford assay calibration curve with BSA protein. Data was fitted to a logarithmic curve: $alog_2(b + Protein) + c$, where a = 0.241, b = 26.231, c = -0.64 are fitting parameters. (**C**) Comparison of protein concentration estimation for the two assays (Bradford and Biuret) and for the results of both combined (Both). Statistical significance of the differences was estimated with a t-test and p-value significance is given.

# 1. TRANSPARENT METHODS

## 1.1. Strains

To investigate chromosomal dynamics, building on previous studies (Javer et al., 2013, 2014; Wlodarski et al., 2017), we used two *E. coli* MG1655 strains with the GFP-ParB/*parS* fluorescent labelling system (kindly provided by Dr Olivier Espéli and Dr Frédéric Boccard (Rocha, 2008)). In each of these strains a P1 *parS* site inserted at either Ori2 or Ter3 positions on the chromosome (3,928,826 and 1,341,067 chromosomal coordinates, respectively). Loci have names assigned according to the MD they belong to. Expression of the ParB-GFP fusion protein is driven by a leaky promoter on the pALA2705 plasmid (Figure S10). No isopropyl $\beta$-D-1-thiogalactopyranoside (IPTG) induction was required to produce the ParB-GFP levels necessary to visualize and track loci.

To investigate cytosolic dynamics we used the *E. coli* CJW4617 strain (kindly provided by Christine Jacobs-Wagner's laboratory) of a MG1655 background capable of expressing $\mu$NS-GFP fusion protein. The avian reovirus protein $\mu$NS is a self-assembling protein (Broering et al., 2002) and its C-terminal fragment can form globular cytoplasmic particles, even when fused to GFP (Broering et al., 2005). $\mu$NS-GFP synthesis is under control of the chromosomal IPTG-inducible promoter lac. In this strain, the lactose/IPTG permease-encoding, lacY gene is deleted from the lac operon. Crucially, $\mu$NS-GFP aggregates are unlikely to make specific interactions with components of the bacterial cytoplasm, given the evolutionary divergence between bacteria and the avian reovirus. We induced the synthesis of the aggregates with 1 mM IPTG for 3.5-4 h, centrifuged the culture at 4,000 rpm for 10 min, and stopped induction by washing the pellet with the growth medium directly before experiments. Induction of GFP-$\mu$NS synthesis usually resulted in a single fluorescent focus per cell.

## 1.2. Culture conditions

All chemical reagents were obtained from Sigma-Aldrich unless otherwise stated. For all microscopy experiments, we used the "minimal medium" consisting of M9 minimal salts (BD) supplemented with complementary salts (CS; $MgSO_4$ 2 mM, $CaCl_2$ 100 $\mu$M, tryptophan 4 $\mu$g/mL, and thymidine 5 $\mu$g/mL), 0.4% glucose (Glu), and 0.5% casamino acids (CAAs; BD). Strains were stored at -80$^o$C in lysogeny broth (LB) +25% glycerol stocks and were streaked on LB plates (containing relevant antibiotic for selection: ampicillin 100 $\mu$g/mL and chloramphenicol 25 $\mu$g/mL). From each plate, bacteria from 3-4 distinct colonies were selected to inoculate the growth medium and strains were grown overnight at 37$^o$C in LB with ampicillin 100 $\mu$g/mL, with shaking at 200 rpm at a 45$^o$ angle. Overnight cultures were diluted 1:200 into 2 mL of fresh minimal medium (with no antibiotic) and grown at 30$^o$C to the optical density at a 600 nm wavelength ($OD_{600}$) of 0.2-0.3 (early exponential growth phase) and transferred onto agarose pads for image acquisition. Doubling times in bulk (at 37$^o$C in the minimal medium with CAAs) were 65 min, Ori2 and Ter3 MG1655; 80 min, CJW4617. For the estimation of cell density, overnight cultures of the CJW4617 strain were diluted 1:200 in 250 ml Luria-Bertani (LB) broth (0.5% yeast extract, 1% Bacto Tryptone, 0.5% NaCl) with no antibiotic and grown at 37$^o$C with 200 rpm shaking. Upon reaching an $OD_{600}$ of 0.1 - 0.3, the various antibiotics and sorbitol were added to the cultures and incubated for further 60 minutes before harvesting for optical density, dry mass and refractive index estimations.

| | Agarose pads | | Liquid (bulk) culture ($IC_{50}$) |
|---|---|---|---|
| | Determined MIC range | Tested concentration (~75% of MIC) | |
| ciprofloxacin | 0.012-0.014 µg/mL | 0.01 µg/mL (0.3 µM) | Ori2: 0.011 µg/mL (0.033 µM)<br>Ter3: 0.016 µg/mL (0.048 µM)<br>µNS: 0.013 µg/mL (0.039 µM) |
| rifampicin | 1.2-1.4 µg/mL | 1.0 µg/mL (1.2 µM) | Ori2: 2.7 µg/mL (3.3 µM)<br>Ter3: 2.4 µg/mL (2.9 µM)<br>µNS: 2.9 µg/mL (3.5 µM) |
| tetracycline | 0.8-1.0 µg/mL | 0.7 µg/mL (1.6 µM) | Ori2: 0.47 µg/mL (1.1 µM)<br>Ter3: 0.75 µg/mL (1.7 µM)<br>µNS: 0.57 µg/mL (1.3 µM) |
| vancomycin | 32-36 µg/mL | 25 µg/mL (17.3 µM) | Ori2: 20.3 µg/mL (14.0 µM)<br>Ter3: 25.7 µg/mL (17.7 µM)<br>µNS: 12.8 µg/mL (8.8 µM) |
| sorbitol | N/A | 72.8 mg/mL (400 mM) | 72.8 mg/mL (400 mM) |

Table S3: **Antibiotic and sorbitol concentrations used in this work, related to Figure 1.** For agarose pad experiments, the MIC range for each antibiotic was determined for each of the three strains (MIC ranges did not differ between different strains). Tested sub-lethal concentrations were ∼75% of the MIC. For the Bradford test, involving measurements on bulk cultures, dose-response curves were constructed (Figure S1) and fitted $IC_{50}$ concentrations were selected for experiments.

## 1.3. Sample preparation for microscopy experiments

Agarose pads contained 1.5% w/v agarose dissolved in the minimal medium and (if required) a fixed concentration of an antibiotic. Pads were approximately 8 mm in diameter and 0.5 mm in thickness. 2.5 $\mu$L of the culture were deposited on a pad under aseptic conditions. The pad was then sealed between a cover slip and a glass slide with a stack of 3 frame seals (Fisher Scientific) to ensure access to excess of oxygen. Under the microscope, the sample was maintained at 30$^o$C during image acquisition for 2 hours, waiting for 20 min before acquiring the first image. Doubling times (without treatment) on agarose pads were 45 min (Ori2 MG1655), 50 min (Ter3 MG1655), and 63 min (CJW4617) (all ± 10 min) measured for individual cells (about 550 cells per strain).

For temperature control, we used a custom built proportional–integral–derivative (PID) temperature controller with two output channels, developed by Dr Jurij Kotar. One channel is used for heating a microscope objective with a heat-

ing collar, the other channel is used for heating a Fluorine doped Tin Oxide (FTO) glass plate. A sample sits on the glass plate. Temperature is measured with K-type thermocouples.

### *1.4. Determining sub-lethal antibiotic concentrations*

In all experiments, we treated bacteria with antibiotic concentrations capable of affecting significantly the cellular physiology but allowing for normal growth. The minimal inhibitory concentration (MIC) range for each antibiotic was determined for each of the three strains (MG1655 Ori2, MG1655 Ter3, and CJW4617 $\mu$NS) using a standard agar dilution MIC determinaton method (Wiegand et al., 2008). Determined MIC ranges did not differ between different strains. Tested sub-lethal concentrations were ~75% of the MIC (Table S3). Sorbitol was tested at a 400 mM concentration, capable of inducing a hyperosmotic shock in *E. coli* as reported previously (Rojas et al., 2014). As agarose pads can absorb liquid, it is possible that dissolved antibiotic was diluted after loading the pad with culture. The volume of an agarose pad was about 25 $\mu$L and the volume of the loaded culture was 2.5 $\mu$L resulting in an up to a $\times$1.1 dilution. However, a smaller dilution is likely as some of the loaded culture evaporated rather then being absorbed into the pad.

Dose response curves were obtained for the Bradford test using a FLUOstar OMEGA 96-well plate reader (BMG Labtech). Bacteria were grown at 30$^o$C after diluting overnight cultures 200:1 into 300 $\mu$L of minimal medium containing a range of antibiotic concentrations, and measuring the $OD_{600}$ every 30 min for 12 h with shaking at 200 rpm. The slopes of the linear parts of the growth curves from at least 4 biological replicates were calculated and the means used to construct the dose-response curves (Figure S1), fitted with Equation S1:

$$g(c) = \frac{g_0}{1 + \frac{c}{IC_{50}}}, \tag{S1}$$

where $g$ is growth rate, $g_0$ is the growth rate without antibiotic, $c$ is antibiotic concentration, and $IC_{50}$ is the fitted $IC_{50}$ concentration, selected for experiments (Table S3).

### *1.5. Image acquisition and processing*

#### *1.5.1. Equipment specifications.*

We used a Nikon Eclipse TiE inverted microscope with a 60$\times$ oil immersion objective (NA=1.45). Images were further magnified with a 2.5$\times$ TV adapter before detection on an Andor iXon EM-CCD camera, capable of detecting single fluorophores and yielding a high signal-to-noise ratio to enable high marker localisation precision. Blue LED with a 470 nm peak and 20 nm spectral width was used to excite the GFP using a Semrock LED-FI filter with the exciter 474 nm (25 nm spectral width), dichroic 495 nm, and emitter 515 nm (25 nm spectral width) bands. Focus during image acquisition was maintained with the Nikon perfect focus system (PFS).

#### *1.5.2. Image acquisition.*

For marker tracking, 21 manually selected fields of view were scanned, each field of view containing about 30 fluorescent markers. 45 s movies were acquired at a 9.6 frame-per-second frame rate with an exposure time of 104 ms. In addition to the movie acquired in the fluoresence mode, a phase contrast image, and a dark frame (acquired immediately before the fluorescent image acquisition using identical settings but no illumination) of each field of view were acquired at every scan. During experiments, each field of view was scanned 6 times, 20 min apart (total scanning time 2 h).

#### *1.5.3. Image processing and data analysis.*

First, the dark frame was subtracted from all other images. For marker dynamics analysis, image processing methods, tracking analysis, image feature extraction, and $MSD$ fitting algorithms were identical to those previously developed and reported by Javer *et al.* (Javer et al., 2013). All image processing and data analysis including plotting the data were accomplished with custom-written programs using in-built and open-source functions of MATLAB® software, with occasional use of Python, ImageJ, IrfanView, and Inkscape.

### *1.6. Semi-manual cell size analysis*

We performed semi-manual measurements of cell lengths and widths. The length of the long and short axis of individual bacteria were measured manually using built-in MATLAB® image display and coordinate marking functions (Figure S13). Cell length was calculated by determining the distance between two furthest points on the bacterium edge (marked on images in Figure S13C). For highest accuracy, all semi-manual measurements were performed by the same person and identical criteria were applied to determine cell boundaries for all measured cells across all treatment conditions.

Semi-manual cell size analysis of phase contrast images was further corroborated with the SuperSegger program designed for automated bacteria segmentation and lineage generation. For SuperSegger software features, capabilities and benchmarks refer to (Stylianidou et al., 2016), while for gating capabilities refer to (Cass et al., 2017).

### *1.7. Dry mass estimation*

Cells grown to an $OD_{600}$ of 0.1-0.3 were treated with antibiotics or sorbitol for 60 minutes as explained above. Their dry mass was estimated as in (Basan et al., 2015). Briefly, sample $OD_{600}$ was recorded from diluted aliquots and the remaining culture centrifuged at 8000g for 10 minutes. The obtained pellet was washed in 200 ml of water, centrifuged again and the pellet re-suspended in 2 ml water. Samples were transferred onto pre-weighted aluminium boats, desiccated overnight at 70$^o$C and weighted.

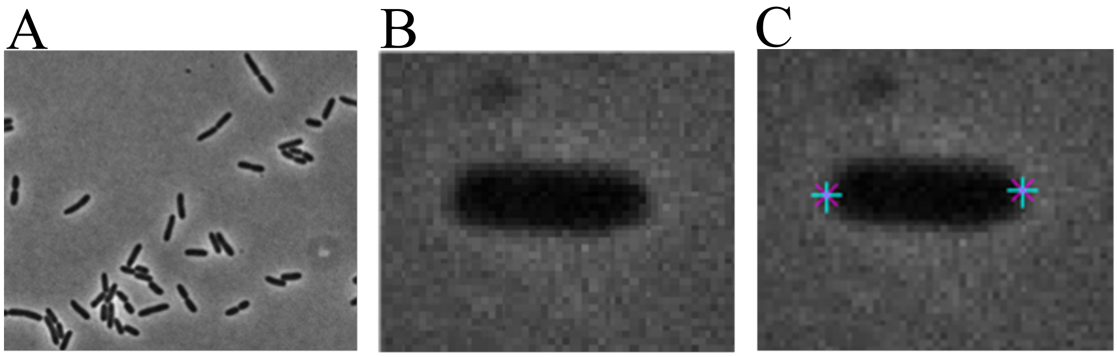


Figure S13: **Semi-manual single-cell level cell size measurements, related to Figure 5.** (**A**) Example images of *E. coli* in the phase contrast mode. (**B**) Images of individual cells were enlarged manually for higher measurement accuracy. (**C**) Length of each measured cell was determined by calculating the distance between two furthest points on the bacterium edge (marked on images with star symbols).

*1.8. Refractive index estimation*

The refractive index (RI) of treated and untreated cells was assayed by comparison with the one of bovine serum albumin (BSA) solutions as in (Marquis, 1973; Bateman et al., 1966). The OD of medium in which cells have been re-suspended matches the one of cell-free medium if medium and cells have the same RI. The addition of BSA changes the RI of solutions by a known amount which scales linearly with BSA concentration (Figure **??**B, inset) (Barer and Tkaczyk, 1954; Crespi et al., 2012), such that the RI of cells can be simply inferred from the concentration of BSA necessary to make their optical density vanish. In our case, BSA (Sigma) was dissolved in water at a final concentration of 10, 20 and 30% in weight. NaCl was supplemented to these solutions in order to match the osmolarity of growth medium and limit artifacts due to osmotic effects that would alter cell size. Aliquots of treated and untreated cells were resuspended in the various BSA solutions, and their $OD_{700}$ estimated.

*1.9. Protein content estimation*

To assay whether the changes in dry mass were due to changes in protein content, protein concentration was examined via two independent colorimetric assays: the Biuret and Bradford assays (see Figure S12, Supplementary Materials). The Biuret assay was carried out as in (You et al., 2013). Samples from treated and untreated cells were washed via centrifugation at 8000g for 2 minutes with 1 ml water, resupended in 0.5 ml water and freezed at -80$^o$C. Samples were thawed at room temperature, 0.25 ml of a 3M NaOH solution were added and boiled at 100$^o$C for 5 minutes. Samples were let to cool down at room temperature and 0.25 ml of 1.6% CuSO4 were added. Solutions were incubated at room temperature for 5 minutes and centrifuged at maximum speed. The optical density of the supernatant was assayed at $\lambda$= 555 nm and protein concentration calculated by comparison with calibration curves obtained by carrying out the assay on known concentrations of BSA. For the Bradford assay (Kruger, 2009), pellet from samples washed in 1 ml water was re-suspended in 0.2 ml of B-PER$^{TM}$ (Thermo Scientific) Bacterial Protein Extraction Reagent and shaken for 15 minutes at room temperature. Samples were then centrifuged at maximum speed for 2 minutes and the supernatant diluted 1:10 into Coomassie stain (Thermo Scientific). After further 10 minutes incubation at room temperature, the optical density of the samples was assayed at $\lambda$= 595 nm and compared to a calibration curve obtained from BSA dilutions.

## References

Barer, R., Tkaczyk, S., 1954. Refractive index of concentrated protein solutions. Nature 173, 821–822.

Basan, M., Zhu, M., Dai, X., Warren, M., Sévin, D., Wang, Y.P., Hwa, T., 2015. Inflating bacterial cells by increased protein synthesis. Mol. Sys. Biol. 11, 836.

Bateman, J.B., Wagman, J., Carstensen, E.L., 1966. Refraction and absorption of light in bacterial suspensions. Kolloid-Zeitschrift und Zeitschrift für Polymere 208, 44–58.

Broering, T.J., Arnold, M.M., Miller, C.L., Hurt, J.A., Joyce, P.L., Nibert, M.L., 2005. Carboxyl-Proximal Regions of Reovirus Nonstructural Protein muNS Necessary and Sufficient for Forming Factory-Like Inclusions. J. Virol. 79, 6194–6206.

Broering, T.J., Parker, J.S.L., Joyce, P.L., Kim, J., Nibert, M.L., 2002. Mammalian Reovirus Nonstructural Protein NS Forms Large Inclusions and Colocalizes with Reovirus Microtubule-Associated Protein 2 in Transfected Cells. J. Virol. 76, 8285–8297.

Cass, J.A., Stylianidou, S., Kuwada, N.J., Traxler, B., Wiggins, P.A., 2017. Probing bacterial cell biology using image cytometry. Mol. Microbiol. 103, 818–828.

Crespi, A., Lobino, M., Matthews, J.C.F., Politi, A., Neal, C.R., Ramponi, R., Osellame, R., O'Brien, J.L., 2012. Measuring protein concentration with entangled photons. Appl. Phys. Lett. 100, 233704.

Javer, A., Kuwada, N.J., Long, Z., Benza, V.G., Dorfman, K.D., Wiggins, P.a., Cicuta, P., Lagomarsino, M.C., 2014. Persistent super-diffusive motion of Escherichia coli chromosomal loci. Nat. Commun. 5, 3854.

Javer, A., Long, Z., Nugent, E., Grisi, M., Siriwatwetchakul, K., Dorfman, K.D., Cicuta, P., Cosentino Lagomarsino, M., 2013. Short-time movement of E. coli chromosomal loci depends on coordinate and subcellular localization. Nat. Commun. 4, 3003.

Kruger, N.J., 2009. The Bradford Method For Protein Quantitation. Humana Press, Totowa, NJ. pp. 17–24.

Marquis, R.E., 1973. Immersion refractometry of isolated bacterial cell walls. J. Bacteriol. 116, 1273–1279.

Rocha, E.P.C., 2008. The organization of the bacterial genome. Annu. Rev. Genet. 42, 211–233.

Rojas, E., Theriot, J.A., Huang, K.C., 2014. Response of Escherichia coli growth rate to osmotic shock. Proc. Natl. Acad. Sci. U.S.A. 111, 7807–7812.

Stylianidou, S., Brennan, C., Nissen, S.B., Kuwada, N.J., Wiggins, P.A., 2016. SuperSegger: robust image segmentation, analysis and lineage tracking of bacterial cells. Mol. Microbiol. 102, 690–700.

Wiegand, I., Hilpert, K., Hancock, R.E.W., 2008. Agar and broth dilution methods to determine the minimal inhibitory concentration (MIC) of antimicrobial substances. Nat. Protoc. 3, 163–75.

Wlodarski, M., Raciti, B., Kotar, J., Cosentino Lagomarsino, M., Fraser, G.M., Cicuta, P., 2017. Both genome and cytosol dynamics change in E. coli challenged with sublethal rifampicin. Phys. Biol. 015005.

You, C., Okano, H., Hui, S., Zhang, Z., Kim, M., Gunderson, C.W., Wang, Y.P., Lenz, P., Yan, D., Hwa, T., 2013. Coordination of bacterial proteome with metabolism by cyclic amp signalling. Nature 500, 301–306.

Yu, S., Sheats, J., Cicuta, P., Sclavi, B., Cosentino Lagomarsino, M., Dorfman, K.D., 2018. Subdiffusion of loci and cytoplasmic particles are different in compressed cells. Communications biology 1, 176.
